# Umbrella Review: Mental Health Interventions for Autistic Children and Adolescents: An Overview of Systematic Reviews and Participatory Equity Synthesis

**DOI:** 10.1016/j.jaacop.2026.02.004

**Published:** 2026-02-26

**Authors:** Alan Lovell, Kieran Becker, Maxwell S. Barnish, Tom Arthur, Joelle Kirby, Helen F. Dodd, Vijaya Raghavan, G.J. Melendez-Torres, Anna Price

**Affiliations:** a University of Exeter, Exeter, United Kingdom; b Schizophrenia Research Foundation, Chennai, India

**Keywords:** adolescent, autism, child, mental health, umbrella review

## Abstract

**Objective:**

Autistic children and adolescents experience high rates of mental health conditions. Objectives were to summarize systematic review evidence and describe quantity and quality of evidence on interventions for the mental health of autistic children and adolescents, how systematic reviews categorize and synthesize intervention effectiveness, how equity-relevant characteristics are addressed, and implications of evidence on equity-relevant characteristics.

**Method:**

MEDLINE, Embase, PsycINFO, and CINAHL databases were searched from 2014 to 2024 to identify systematic reviews on mental health conditions in autistic children and adolescents. After screening of 4,027 records, 31 reviews were included. Data were extracted and assessed in terms of intervention effectiveness and in relation to equity-relevant characteristics. Participatory synthesis was guided by a research advisory group comprising people with lived experience of autism, whose perspectives were incorporated into multiple stages of the research process.

**Results:**

The intervention with the best evidence was cognitive-behavioral therapy, which was effective for all mental health outcomes covered. There was also weak evidence for mindfulness (for depression and anxiety), social skills training (for depression and anxiety) and pharmacological therapies (for anxiety, obsessions, and compulsions). Most reviews were rated as being of low or critically low quality. With few exceptions, reviews did not address equity dimensions of interventions in depth.

**Conclusion:**

Current evidence does not highlight the equity dimensions that impact the effectiveness of mental health interventions for autistic children and adolescents. Future work should take an intervention-wide, equity-deep approach to synthesis and to design and analysis of primary trials.

**Study registration information:**

Mental health interventions for autistic children and adolescents: overview of systematic reviews and participatory equity synthesis; https://www.crd.york.ac.uk/PROSPERO/view/CRD42024612863.

## Plain language summary

This study reviewed interventions for children and adolescents with autism and how equity-relevant characteristics are addressed in these interventions. The authors included 31 studies in this review. This study found that cognitive behavioral therapy (CBT) had the best evidence across all mental health outcomes reviewed. However, included reviews did not adequately assess how personal and social factors impact how interventions work across groups.

There are an estimated 28.3 million autistic people worldwide (369.4 per 100,000 population), although prevalence estimates vary based on diagnostic criteria, regional factors, and evolving awareness.^1^ Autistic individuals (for consideration of language preferences, see Kenny *et al.*^2^ and Keating *et al.*^3^) frequently experience difficulties in social communication and interaction as well as restricted, repetitive interests and behaviors.^2^^,^^3^ They can also experience a range of mental health challenges, and rates of diagnosable mental health conditions are higher in autistic people than in the general population. For example, the prevalence of anxiety disorders and depressive disorders (across all ages) has been estimated to be 20% and 11%, respectively, in the autistic population vs 15% and 8% in the general population.^4^

Mental health challenges in autism often arise in childhood and hence may be amenable to early intervention.^4^, ^5^, ^6^ There have been many studies of mental health interventions—both for prevention and for treatment—in autistic children and adolescents covering a wide range of approaches.^7^^,^^8^ These approaches range from cognitive-behavioral therapy (CBT) and social skills training to pharmacological and dietary interventions. A plethora of systematic reviews have assessed this evidence base, but the focus of these reviews is disparate, and to our knowledge there is no umbrella review that brings this evidence together (2 previous umbrella reviews of autism^9^^,^^10^ did not focus on mental health outcomes).

A key consideration for this field is the extent to which interventions are inclusive. Individuals with certain equity-relevant characteristics may be excluded from interventions and from related research.^11^ Equity-relevant characteristics are characteristics of children and adolescents, their families, or where they live that impact how effective health services are for them. These include core social determinants of health, such as those identified in the PROGRESS-Plus^12^ heuristic (eg, race/ethnicity, gender, socioeconomic status) alongside more specific relevant characteristics (eg, characteristics of autism spectrum conditions such as minimally verbal, age transitions between adolescent and adult services), and broader contextual and health system differences, such as low-income vs high-income country contexts. The extent to which equity-relevant characteristics are considered within existing systematic reviews of mental health interventions for autistic children and adolescents is unclear, despite the potential of implementation of mental health interventions to exacerbate existing differences.^13^

Our focus for this review is on interventions for the prevention and treatment of mental health conditions for autistic children and adolescents. We include studies that focus on symptoms of mental health conditions (eg, low mood), but not studies that focus on intermediate constructs (eg, self-esteem). The focus is not on interventions (or reviews of interventions) that target functional impairments or other issues related mainly to autism as a diagnostic category. We undertook this systematic review using a participatory synthesis strategy, supported by an involvement and engagement group of autistic adolescents and parents of autistic children and adolescents. Our research questions are as follows:

1. What is the quantity and quality of systematic review evidence on pharmacological and nonpharmacological interventions for the mental health of autistic children and adolescents (ie, interventions that have an improvement in mental health as their objective, such as change in depression or anxiety scores)?
2. How do these systematic reviews categorize and synthesize intervention effectiveness, and what are the gaps in structuring evidence?
3. How are equity-relevant characteristics currently addressed in such systematic reviews and, where appropriate, in evidence within reviews, and which characteristics are missing?
4. What are the implications of the described evidence regarding equity-relevant characteristics for research, policy, and practice?

## Method

### Study Design and Search

An umbrella review methodology was employed, synthesizing existing systematic reviews relating to mental health interventions for autistic children and adolescents. A protocol for the study was preregistered on PROSPERO (CRD42024612863), which specified the search strategy, eligibility criteria, and methods of data extraction and synthesis used in our analyses. Research question 3 of the protocol (“What are the equity-relevant characteristics of children and adolescents and their contexts that are relevant to intervention effectiveness and implementation?”) will be covered in a companion commentary.

We searched MEDLINE, Embase, APA PsycINFO (Ovid), and CINAHL (EBSCO) databases from 2014 until November 8, 2024, to identify systematic reviews of mental health conditions in autistic children and young people. Unpublished studies were not sought, and there was no grey literature search. The full search strategy is shown in Supplement 1, available online.

Systematic reviews published as full texts in the last 10 years (2014 onward) that aimed at evaluating effectiveness or implementation of relevant interventions were included. Reviews were included whether or not they incorporated meta-analyses or formal grading of evidence. Reviews that did not state specific search strategies, provide inclusion and exclusion criteria, or state a method for synthesizing identified evidence were excluded. Scoping reviews were also excluded, as were systematic reviews that synthesized a broad range of evidence types beyond the scope of effectiveness and implementation evidence in a way that could not be separated.

### Participants and Population

We included reviews that focused on autistic children and adolescents (up to 19 years of age) or that presented results for autistic children and adolescents as a distinct subgroup of findings. As we examined review-level evidence, we did not exclude reviews based on the specific terms of clinical diagnosis (autistic spectrum conditions/autism spectrum disorder [ASD], Asperger’s syndrome, and pervasive development disorder not otherwise specified all were included) or the verification tools used in included studies within reviews (eg, whether standardized assessments had been performed, such as the Autism Diagnostic Observation Schedule [ADOS] or Autism Diagnostic Interview–Revised [ADI-R]). We included reviews on neurodiverse populations more broadly in which a subgroup focused on autistic children and adolescents or on children and adolescents with autistic traits was included.

### Interventions and Comparators

Mental health interventions were defined as interventions evaluated for prevention or treatment of mental health problems. Interventions implemented with parents or carers were eligible if outcomes relating to mental health of children and adolescents were included. Any comparators, including standard care or waitlist control, were included.

### Main Outcomes

For reviews focusing on effectiveness, we considered any outcome associated with mental health–related symptoms or diagnoses. Review findings were classified by intervention type followed by outcomes studied. For reviews focusing on implementation, we considered data relating to acceptability, feasibility, and mechanisms of interventions and their implementation. We excluded reviews focusing on symptoms of autism as an outcome or capturing quality-of-life impacts more broadly.

### Data Extraction and Risk of Bias Assessment

The review was managed using EPPI Reviewer 6.^14^ Identified records were deduplicated and screened at title and abstract. Potentially eligible abstracts were taken forward to full text and screened by 2 reviewers working independently and in duplicate. Disagreements were resolved with recourse to a third reviewer.

A machine learning classifier in EPPI Reviewer 6 was used to assign each record a score, recording how likely it was to be a systematic review. Records scoring less than 20% were single screened, and records scoring 20% or above were double screened at title and abstract.

Data extracted from each review included the review aims and PICO (patient or problem, intervention, comparison, outcome); number of relevant included studies; and key findings, including any evidence from relevant subgroup analyses or relating to equity-relevant characteristics. Data were extracted by 1 reviewer into a standardized form on EPPI Reviewer 6 and checked by a second reviewer.

Two reviewers appraised the quality of reviews using AMSTAR-2 in duplicate and independently. Disagreements were resolved with recourse to a third reviewer. We followed the guidance notes available for each question on the AMSTAR Checklist.^15^^,^^16^

### Data Synthesis

We developed a set of labels that captured intervention characteristics and assigned them to included studies, noting that studies may assess more than 1 intervention type. Subsequently, we assessed the evidence landscape for different combinations of intervention type and mental health outcomes both at the individual review level and across the evidence base. We explored equity characteristics assessed by included reviews and what insights these provided, using this to generate summary of findings tables for both effectiveness and equity. In considering the conclusions of the body of evidence, we studied the quality profile of included reviews as assessed by AMSTAR-2.

To address our equity-focused research questions, we narratively synthesized data that captured how some aspect of an intervention (eg, its implementation or effectiveness) related to an equity-relevant characteristic. Here, we specifically drew upon both review-level foci (eg, reviews focused on autistic children and adolescents in low- and middle-income countries [LMICs]) and equity analyses within reviews (eg, subgroup analyses by gender, by race/ethnicity, or by disability), to develop a descriptive account of how mental health interventions impact health disparities within autistic children and adolescents. Extracted themes were deductively categorized based on their specific equity characteristics, with review-level and within-review data synthesized for each type to intervention to explain how and why they may be equity or inequity generating at an individual, group, and/or service level.

### Participatory Synthesis

The review benefitted from our research team including autistic and nonautistic researchers, as well as involvement and engagement activities conducted in accordance with National Institute for Health Research (NIHR) guidelines.^17^ A research advisory group (RAG) was recruited, which consisted of 4 people with lived experience of ASD, including an autistic young person and parents/carers. The RAG was recruited on a rolling basis via preestablished researcher networks linked to the Children and Young People’s Mental Health Research Collaboration (ChYMe)^18^ and Science of ADHD and Neurodevelopment Collaboration (SAND)^19^ at the University of Exeter. The aim of the RAG was to integrate perspectives of people with lived experience into multiple stages of the research process, including consultation of the protocol and interpretation of the results. The RAG was consulted via 3 focus group meetings, and a series of one-on-one meetings (participants were given the choice to engage as group or individually) conducted by 2 authors (K.B. and A.P.). Initial meetings introduced RAG members to the research, involved general discussions of the study, and included 1 member reviewing the protocol and language used.

After preliminary results, we consulted the RAG again to develop a set of intervention characteristics identified as being important and then extracted them from the data. Finally, we worked with our RAG to interpret our findings for both the intervention characteristics and the equity-relevant characteristics, with a focus on addressing equity-relevant characteristics that have not yet been researched or synthesized. Patient and public involvement and engagement (PPIE) insights in relation to the aims and findings of this umbrella review were recorded throughout and will be presented in a companion commentary.

## Results

### Search Results

**Figure 1:**
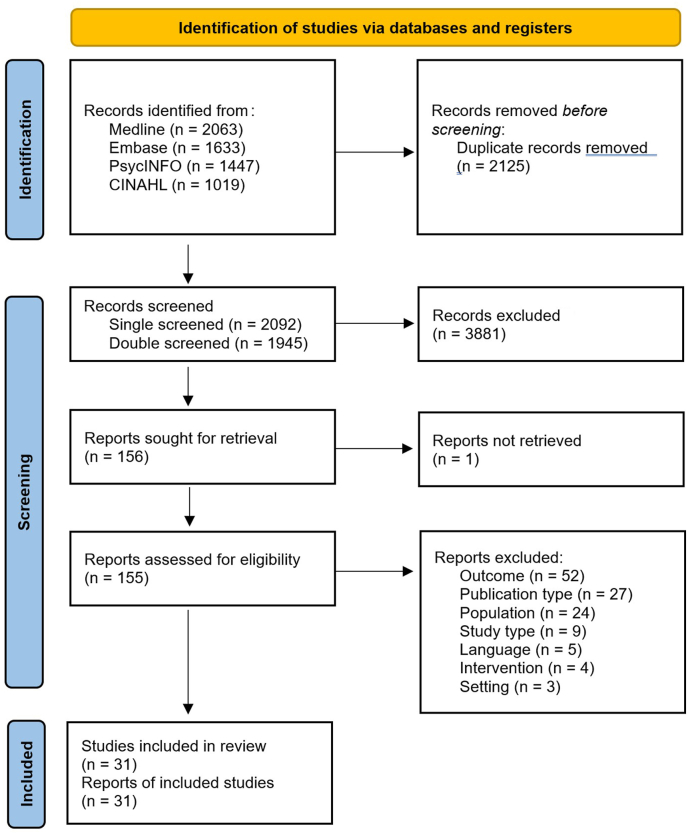
PRISMA (Preferred Reporting Items for Systematic reviews and Meta-Analyses) Flowchart ***Note:****Please note color figures are available online.*

Our searches of Medline, Embase, PsycINFO, and CINAHL retrieved 6,162 records. After deduplication, this was reduced to 4,027 records. These records were run through the classifier in EPPI Reviewer 6;records classified as having less than a 20% chance of being a systematic review were single-screened by title and abstract (2,092 records), and the remaining 1,945 records were double-screened. After title and abstract screening, 156 records proceeded to full text screening, at which stage 125 records were excluded (see Tables S1 and S2, available online, for the inclusion criteria and a list of titles excluded at full text screening, respectively). This left 31 records for analysis, all of which originated from the double-screen batch at title and abstract screening. A PRISMA (Preferred Reporting Items for Systematic reviews and Meta-Analyses) flowchart is shown in Figure 1.

### Included Reviews

Included reviews were published between 2014 and 2024. Eighteen reviews included a meta-analysis. Table 1 summarizes characteristics of each review^8^^,^^20^, ^21^, ^22^, ^23^, ^24^, ^25^, ^26^, ^27^, ^28^, ^29^, ^30^, ^31^, ^32^, ^33^, ^34^, ^35^, ^36^, ^37^, ^38^, ^39^, ^40^, ^41^, ^42^, ^43^, ^44^, ^45^, ^46^, ^47^, ^48^, ^49^; settings and sociodemographic factors for included studies are reported in Table 2.

**Table 1:** Summary of Included Reviews

First author, year	Focus of review	Type of review	Range of included studies, y	No. of studies included	Participants in review	Intervention type
Cameron, 2021^20^	Psychological interventions for depression in children and young people with an intellectual disability and/or ASD	Narrative systematic review	1982-2019	10	190 participants; ages 3-25 y; ∼60%-80% male when reported	CBT/adapted CBT/CBT-based/ACT; behavioral/behavior training/behavior therapy/ABA; psychotherapy NOS; physical activity
Chen, 2022^21^	XR and telehealth interventions for children and adolescents with ASD	Systematic review (no meta-analyses)	2010-2022	112 (2 trials focused on anxiety reduction)	3,825 children and adolescents diagnosed with ASD; ages 1-19 y; gender not reported	Technology based
D’Alo, 2021^22^	Management of ASD, with focus on use of antipsychotics in children and adolescents	Systematic review and meta-analysis	1978-2017	21 (1 RCT focused on anxiety and 4 on OCD)	1,309 children and adolescents with ASD; ages 0-18 y; ∼70%-95% male, when reported	Pharmacological
De Crescenzo, 2020^23^	Polyunsaturated fatty acids in children and adolescents with ASD	Systematic review and meta-analysis	2007-2018	9 (1 RCT focused on anxiety)	405 participants; mean age 6.7 y; 84% male	Biomedical
De Vries, 2015^24^	Therapeutic use of music as an intervention with children with ASD	Narrative systematic review	2001-2014	15 (1 study focused on anxiety)	91 participants for primary studies; 2 reviews were also included; ages 2-9 y; boys majority of participants	Arts-based (music, art, theater)
Francis, 2022^25^	Play-based interventions on mental health outcomes in children with DLD and ASD	Systematic literature review with meta-analysis	2009-2021	10	469 children with ASD; ages 2-16 y; 82% boys; interventions were more frequently implemented with participants <12 y (7 studies)	Play-based
Gregus, 2023^26^	CBT to address symptoms of depression in autistic individuals	Systematic review with meta-analysis	2008-2023	28	631 treatment group participants; average age range 10-49 y; where reported, 46%-88.2% male	CBT/adapted CBT/CBT-based/ACT
Gupta, 2023^27^	Buspirone in core symptoms of ASD and co-occurring anxiety	Systematic review	1989-2019	6 (2 studies focused on ASD)	268 children with ASD with co-occurring anxiety; ages 6-17 y; gender not reported	Pharmacological
Hillman, 2020^28^	Interventions for reducing anxiety in school-age children with ASD	Systematic review and meta-analysis	2005-2018	24	931 children with ASD; ages 3-19 y; 82% boys; most studies restricted to participants with functioning above a certain level of cognitive ability	CBT/adapted CBT/CBT-based/ACT; arts-based (music, art, theater); relaxation/mind-body/massage
James, 2020^29^	CBT for childhood anxiety disorders	Systematic review with meta-analysis	2007-2018	87 (12 focused on autistic children)	5,964 children in quantitative analysis; up to 19 y of age; summary age and gender statistics not reported	CBT/adapted CBT/CBT-based/ACT
Kose, 2018^30^	CBT for individuals who have comorbid ASD and OCD	Systematic review	2003-2017	11	170 children, adolescents, and adults; children ages 7-18; 65% male; all had high-functioning autism and IQ >69	CBT/adapted CBT/CBT-based/ACT
Kreslins, 2015^31^	Psychosocial interventions to manage anxiety in children and adolescents with ASD	Systematic review with meta-analysis	2005-2013	10	470 autistic participants with clinically significant anxiety symptoms; ages 7-17 y; 393 males, 72 females, and 5 not reported	CBT/adapted CBT/CBT-based/ACT; social skills
Lee, 2024^32^	Culturally responsive autism interventions among minoritized autistic children and their families	Systematic review (with meta-analysis)	2014-2023	24	807 caregivers and 759 children, across a range of ages and cultures; ages 0-18 y for children; gender not reported	Social skills; parent management/parent training/parent psychoeducation/family therapy; CBT/adapted CBT/CBT-based/ACT; behavioral/behavior training/behavior therapy/ABA
Linden, 2023^8^	Interventions to improve mental health of autistic people	Systematic review and network meta-analysis	1993-2020	71	3,630 participants; 17 studies were in adults, 1 was in young adults ages 18-25 y, all others were in children; gender not reported	CBT/adapted CBT/CBT-based/ACT; pharmacological; arts-based (music, art, theater); parent management/parent training/parent psychoeducation/family therapy; psychotherapy NOS; behavioral/behavior training/behavior therapy/ABA; social skills
Liu, 2024^33^	Physical activity interventions for mental health in children and adolescents with neurodevelopmental disorders	Systematic review and meta-analysis	1995-2023	76 (19 focused on autistic children)	3,007 participants; age range typically primary or primary-to-secondary school; most studies 70%-100% male	Physical activity
Loftus, 2023^34^	Mindfulness-based therapy for anxiety, social behaviors, and aggressive behaviors in children with ASD	Narrative systematic review	2010-2021	23	436 children with ASD; ages 5-17 y; 81.25% male among studies that reported gender	Mindfulness-based
Perihan, 2020^35^	CBT to reduce anxiety-related outcomes in children with high-functioning ASD	Systematic review and meta-analysis	Search date not stated	23	745 children; ages 5-18 y; 82.6% male among studies that reported gender	CBT/adapted CBT/CBT-based/ACT
Perihan, 2022^36^	School-based anxiety treatments for children with ASD	Systematic review and meta-analysis	2008-2019	6	165 participants; ages 5-21 y; 95 males and 70 females	CBT/adapted CBT/CBT-based/ACT; social skills
Riis, 2025^37^	Physical activity to reduce anxiety in autistic people	Systematic review (no meta-analysis)	2009-2022	8	304 participants; ages 5-27 y; 47 females and 145 males; gender not reported in 130; 3 studies included people with co-occurring conditions (eg, ADHD, OCD)	Physical activity
Rosenau, 2026^38^	Psychotherapy for autistic youth	Meta-analysis	2005-2021	29	1,464 participants; ages 4-18 y; 85% male	CBT/adapted CBT/CBT-based/ACT; behavioral/behavior training/behavior therapy/ABA; animal-assisted therapy; arts-based (music, art, theater); play-based interventions
Rumball, 2019^39^	Outline current state of the field of research into PTSD in ASD	Systematic review	1993-2017	24	674 participants with PTSD and ASD or PDD-NOS; ages 6-45 y; 76.5% male	CBT/adapted CBT/CBT-based/ACT; psychotherapy NOS; EMDR; behavioral/behavior training/behavior therapy/ABA
Rumney, 2017^40^	Social skills interventions to influence mood in autistic children and young people	Systematic review (no meta-analysis)	1987-2015	10	399 children and young people; ages 7-18 y; 87% male	Social skills
Sharma, 2021^41^	CBT for reducing anxiety in autistic youth	Systematic review and meta-analysis	2005-2020	19	487 autistic participants diagnosed with co-occurring anxiety disorder; when reported, average age range ∼5-15 y; most studies 60%-100% male	CBT/adapted CBT/CBT-based/ACT
Simione, 2024^42^	Mindfulness-based interventions for autistic individuals and their caregivers	Systematic review (no meta-analysis)	2013-2023	37 (6 focused on children or adolescents, 5 involved youth and parents)	247 participants from youth-only studies; ages and genders not reported	Mindfulness-based
Syriopoulou-Delli, 2024^43^	Therapeutic interventions for mental health conditions in children or adolescents with ASD	Systematic review (no meta-analysis)	2003-2020	15	1,959 children and adolescents with ASD; ages 5-18 y; gender not reported	CBT/adapted CBT/CBT-based/ACT; technology based
Ung, 2015^44^	To examine if treatment efficacy varies as a function of the reporter and treatment modality	Systematic review and meta-analysis	2005-2014	14	511 participants; ages 7-17 y; 83.6% male among studies that reported gender	CBT/adapted CBT/CBT-based/ACT
Vasa, 2014^45^	Psychopharmacological and nonpsycho pharmacological treatments for anxiety in youth with autism	Systematic review	1998-2013	15	516 children and adolescents with ASD and anxiety; ages 4-18 y; gender not reported; all participants in CBT trials were high functioning	CBT/adapted CBT/CBT-based/ACT; pharmacological; sensory interventions; behavioral/behavior training/behavior therapy/ABA
Wang, 2021^46^	CBT on symptoms of ASD and social-emotional problems in children or adolescents with ASD	Systematic review and meta-analysis	1995-2019	51	2,485 children or adolescents with ASD; mean age ∼5-15 y; gender not reported	CBT/adapted CBT/CBT-based/ACT
Warwick, 2017^47^	CBT for childhood anxiety disorders in terms of absence of all anxiety disorders	Meta-analysis	2007-2015	19 (5 studies with autistic people)	1,085 participants with a clinically diagnosed anxiety disorder; autism studies included 349 participants (ages 7-16 y; gender not reported)	CBT/adapted CBT/CBT-based/ACT
Weitlauf, 2014^48^	Behavioral, educational, family, medical, allied health, and CAM treatments for children ages 2-12 with ASD	Narrative systematic review	Study date range not reported	159	3,065 (behavioral), 443 (educational), 2,603 (medical), 518 (allied health), 190 (CAM); ages 2-12 y; no summary details regarding gender provided	CBT/adapted CBT/CBT-based/ACT; pharmacological; behavioral/behavior training/behavior therapy/ABA; environmental; developmental/educational; social skills; play-based interventions; parent management/parent training/parent psychoeducation/family therapy; environmental; technology based; biomedical; relaxation/mind-body/massage
Wichers, 2023^49^	Psychotherapy for comorbid symptoms of anxiety, depression, or OCD in children and adults with ASD	Systematic review and meta-analysis	2012-2021	26 (7 were adult studies)	1,251; ages 5-65 y with mean age typically ∼10-11 y; when reported, studies typically had a large male majority	CBT/adapted CBT/CBT-based/ACT; social skills

Note: ABA = applied behavior analysis; ACT = acceptance and commitment therapy; ADHD = attention-deficit/hyperactivity disorder; ASD = autism spectrum disorder; CAM = complementary and alternative medicine; CBT = cognitive-behavioral therapy; DLD = developmental language disorder; EMDR = eye movement desensitization and reprocessing; NOS = not otherwise specified; OCD = obsessive-compulsive disorder; PDD = pervasive development disorder; PTSD = posttraumatic stress disorder; RCT = randomized controlled trial; XR = extended reality.

**Table 2:** Equity-Relevant Characteristics and Findings That Were Reported in Relation to Mental Health Interventions for Autistic Children and Adolescents

Intervention type	Studied characteristics	Summary of findings
Behavioral therapies	Culture^32^	Culturally responsive interventions found to generally promote positive outcomes
CBT- or ACT-based	Place of residence^29^	Study data almost exclusively from high-income countries
	Race/ethnicity and culture^31^^,^^32^^,^^36^^,^^47^	Limited representation of ethnic minorities (or lack of details reported); culturally responsive interventions promote positive outcomes (comparable to original interventions)
	Gender/sex^31^	Limited representation of females identified within trial-level evidence
	SE status^47^	Demographic data not consistently reported, precluding any evaluations of SE groups
	Disability^29^^,^^30^^,^^43^^,^^45^	Data lacking from children with intellectual disabilities and/or from those who find verbal communication difficult
	Age^41^^,^^43^	Analyses revealed larger effect sizes in younger children; adaptations of CBT for younger children require development
Mindfulness-based	No equity-relevant data synthesized	N/A
Psychotherapy NOS	No equity-relevant data synthesized	N/A
Pharmacological	No equity-relevant data synthesized	N/A
Biomedical	No equity-relevant data synthesized	N/A
Physical activity	Ethnicity/race^37^	Ethnicity/race of participants rarely reported in the trial-level evidence
Family-based	Culture^32^	Culturally responsive interventions found to promote positive outcomes (comparable to original, nonadapted interventions)
Social skills	Place of residence^40^	Studies have been conducted across several countries, including diverse backgrounds
	Race/ethnicity and culture^31^^,^^32^^,^^36^	Limited representation of ethnic minorities identified; culturally responsive interventions found to generally promote positive outcomes (comparable to original, nonadapted interventions)
	Gender/sex^31^	Limited representation of females identified in the trial-level evidence
	Disability^40^	Data lacking from children with intellectual disabilities
Play-based	No equity-relevant data synthesized	N/A
Technology based	Place of residence^21^	Interventions require strong e-communication networks, especially for rural areas
	SE factors^21^	Cost burdens/issues identified (eg, expensive equipment and advanced digital skills needed)
	Age^21^	Outcomes may vary according to different age groups (though not formally examined)
Other interventions	No equity-relevant data synthesized	N/A

Note: ACT = acceptance and commitment therapy; CBT = cognitive-behavioral therapy; N/A = not applicable; NOS = not otherwise specified; SE = socioeconomic.

Nineteen reviews focused exclusively on children and adolescents with ASD. Two reviews^25^^,^^33^ included children and adolescents with ASD and 1 or more other conditions (such as children with intellectual disability or non-ASD neurodivergence), and 2 reviews^29^^,^^47^ included all children and adolescents and included subgroup analyses of participants with ASD. The remaining 8 reviews^8^^,^^20^^,^^26^^,^^30^^,^^37^^,^^39^^,^^42^^,^^49^ included adults as well as children and adolescents but presented subgroup analyses for participants younger than age 18. The reviews covered a wide range of intervention types, which we clustered into 20 categories (Table S3, available online). The most common intervention types were CBT/adapted CBT/CBT-based/acceptance and commitment therapy (ACT) (20 reviews), behavioral/behavior training/behavior therapy/applied behavior analysis (7 reviews), and social skills interventions (7 reviews). A wide variety of comparators and outcomes were reported.

AMSTAR-2 ratings for each of the included reviews are described in Table S4, available online. Quality indicators were highly variable among the reviews, but common limitations were evident. Of the 31 included reviews, 26 did not report the funding sources of included studies, 23 did not register a protocol before work started on the review, and 22 did not provide a list of excluded studies. Other common areas of weakness according to AMSTAR-2 were the fact that only 13 of the included studies sufficiently explained their selection of the study designs for inclusion in the review, and just 14 studies developed their research questions and inclusion criteria using PICO components. Only 2 of the reviews were appraised as being of moderate quality,^8^^,^^29^ 7 were deemed to be of low quality, and the remaining 22 were deemed to be of critically low quality.

During analysis, as similar patterns of results emerged between reviews of low quality and reviews of critically low quality, limited distinction is made between these data in the following sections. However, comparisons between data of moderate quality and low/critically low quality are made hereafter, where possible.

### Effectiveness Outcomes

Table 3 outlines the effectiveness evidence for combinations of specific intervention types and outcomes available within the included reviews. The number of included studies within the reviews ranged from 1 to 159, though some reviews were broader than our scope. The largest review solely on mental health outcomes in autistic people contained 71 studies, of which 53 comprised child populations.^8^ Among the 31 included reviews, 10 (32%)^8^^,^^22^^,^^23^^,^^29^^,^^31^^,^^38^^,^^41^^,^^46^^,^^47^^,^^49^ included only randomized controlled trials, whereas the other reviews included a broader range of study designs, and the proportion of randomized controlled trials was in many instances fairly low, for example, 2 of 8 studies (25%) in Riis *et al.*^37^ and 3 of 10 studies for the efficacy review in Cameron *et al.*^20^ No included reviews excluded studies based on study duration.

**Table 3:** Effectiveness Evidence That Was Reported in Relation to Mental Health Interventions for Autistic Children and Adolescents

Intervention type	Studied outcomes	Summary of findings
Behavioral therapies (relevant studies: Linden, 2023^8^; Cameron, 2021^20^; Lee, 2024^32^; Rosenau, 2026^38^; Rumball, 2019^39^; Vasa, 2014^45^; Weitlauf, 2014^48^)	Anxiety	Two reviews^45^^,^^48^ found weak evidence for a benefit of behavioral therapies on anxiety.
	Depression	Three reviews^8^^,^^20^^,^^48^ found weak evidence for a benefit of behavioral therapies on depression.
	Obsessions and compulsions	One review^48^ found weak evidence for a benefit of behavioral therapies on obsessions and compulsions.
	Overall mental health	Two reviews^32^^,^^38^ assessed the impact of behavioral therapies on overall mental health. Rosenau^38^ found a small effect size.
	PTSD	One review^39^ assessed the impact of behavioral therapies on PTSD and found preliminary evidence of benefit based on 1 small uncontrolled trial.
	Self-harm	None
CBT- or ACT-based (relevant studies: Linden, 2023^8^; Cameron, 2021^20^; Gregus, 2023^26^; Hillman, 2020^28^; James, 2020^29^; Kose, 2018^30^; Kreslins, 2015^31^; Lee, 2024^32^; Perihan, 2020^35^; Perihan, 2022^36^; Rosenau, 2026^38^; Rumball, 2019^39^; Sharma, 2021^41^; Syriopoulou-Delli, 2024^43^; Ung, 2015^44^; Vasa, 2014^45^; Wang, 2021^46^; Warwick, 2017^47^; Weitlauf, 2014^48^; Wichers, 2023^49^)	Anxiety	Evidence from multiple reviews was generally supportive of a benefit of CBT- or ACT-based interventions on anxiety.
	Depression	Four reviews^20^^,^^26^^,^^48^^,^^49^ broadly supported a benefit for CBT- or ACT-based interventions on depression.
	Obsessions and compulsions	Two reviews^30^^,^^48^ generally supported a benefit of CBT- or ACT-based interventions on obsessions and compulsions.
	Overall mental health	Four reviews^32^^,^^38^^,^^43^^,^^46^ assessed the impact of CBT- or ACT-based interventions on overall mental health, and all found evidence of a benefit.
	PTSD	One review^39^ assessed the impact of CBT- or ACT-based interventions on PTSD and found preliminary evidence of benefit.
	Self-harm	None
Mindfulness-based (Loftus, 2023^34^; Simione, 2024^42^)	Anxiety	Two reviews^34^^,^^42^ assessed the impact of mindfulness-based interventions on anxiety, and both supported a benefit.
	Depression	One review^42^ assessed the impact of mindfulness-based interventions on depression and found a benefit.
	Obsessions and compulsions	None
	Overall mental health	None
	PTSD	None
	Self-harm	None
Psychotherapy NOS (relevant studies: Linden, 2023^8^; Cameron, 2021^20^; Rumball, 2019^39^)	Anxiety	None
	Depression	Two reviews^8^^,^^20^ assessed the impact of psychotherapy NOS on depression and generally support a benefit, although there is uncertainty in Cameron’s review^20^ due to methodological issues in included studies.
	Obsessions and compulsions	None
	Overall mental health	None
	PTSD	One review^39^ assessed the impact of psychotherapy NOS on PTSD and generally found evidence of a benefit.
	Self-harm	None
Pharmacological (relevant studies: Linden, 2023^8^; D’Alo, 2021^22^; Gupta, 2023^27^; Vasa, 2014^45^; Weitlauf, 2014^48^)	Anxiety	Five reviews^8^^,^^22^^,^^27^^,^^45^^,^^48^ assessed the impact of pharmacological interventions on anxiety. Across reviews, there was some evidence for a benefit, but it was not consistent across trials, and there were concerns about maintenance of benefit.
	Depression	Two reviews^8^^,^^48^ assessed the impact of pharmacological interventions on depression, and the results were overall inconclusive.
	Obsessions and compulsions	One review^48^ found evidence for a benefit of certain types of pharmacological intervention on behaviors including obsessions and compulsions.
	Overall mental health	One review^22^ assessed the impact of pharmacological interventions on overall mental health and found evidence of a benefit.
	PTSD	None
	Self-harm	None
Biomedical (relevant studies: De Crescenzo, 2020^23^; Weitlauf, 2014^48^)	Anxiety	Two reviews^23^^,^^48^ assessed the impact of biomedical interventions on anxiety. The former found evidence to support benefit, but there was very low certainty.
	Depression	One review^48^ assessed the impact of biomedical interventions on depression and found no clear evidence of benefit.
	Obsessions and compulsions	One review^48^ assessed the impact of biomedical interventions on obsessions and compulsions and found no clear evidence of benefit.
	Overall mental health	One review^23^ assessed the impact of biomedical interventions on overall mental health and found no evidence of benefit.
	PTSD	None
	Self-harm	One review^23^ assessed the impact of biomedical interventions on self-harm, and evidence of benefit was unclear.
Physical activity (relevant studies: Cameron, 2021^20^; Liu, 2024^33^; Riis 2025^37^)	Anxiety	One review^37^ assessed the impact of physical activity on anxiety and found evidence of benefit.
	Depression	One review^20^ assessed the impact of physical activity on depression and found limited evidence of a small benefit from an assisted cycling study.
	Obsessions and compulsions	None
	Overall mental health	One review^33^ assessed the impact of physical activity on overall mental health and found evidence of benefit.
	PTSD	None
	Self-harm	None
Family-based (relevant studies: Linden, 2023^8^; Lee, 2024^32^; Weitlauf, 2014^48^)	Anxiety reduction	Two reviews^8^^,^^48^ assessed the impact of family-based interventions on anxiety and found no clear evidence of additional benefit.
	Depression	Two reviews^8^^,^^48^ assessed the impact of family-based interventions on depression and found no clear evidence of additional benefit.
	Obsessions and compulsions	One review^48^ assessed the impact of family-based interventions on obsessions and compulsions and found no clear evidence of benefit.
	Overall mental health	One review^32^ assessed the impact of family-based interventions on overall mental health and found evidence of benefit.
	PTSD	None
	Self-harm	None
Social skills (relevant studies: Linden, 2023^8^; Kreslins, 2015^31^; Lee, 2024^32^; Perihan, 2022^36^; Rumney, 2017^40^; Weitlauf, 2014^48^; Wichers, 2023^49^)	Anxiety	Five reviews^31^^,^^36^^,^^40^^,^^48^^,^^49^ assessed the impact of social skills interventions on anxiety and typically found consistent evidence of benefit.
	Depression	Four reviews^31^^,^^40^^,^^48^^,^^49^ assessed the impact of social skills interventions on depression and typically found consistent evidence of benefit.
	Obsessions and compulsions	One review^48^ assessed the impact of social skills interventions on behavior including obsessions and compulsions and found some evidence of benefit.
	Overall mental health	One review^32^ assessed the impact of social skills on overall mental health and found evidence of benefit.
	PTSD	None
	Self-harm	None
Play-based (relevant studies: Francis, 2022^25^; Rosenau 2026^38^; Weitlauf, 2014^48^)	Anxiety	Two reviews^25^^,^^48^ assessed the impact of play-based interventions on anxiety, and 1 review^25^ found evidence of benefit.
	Depression	Two reviews^25^^,^^48^ assessed the impact of play-based interventions on depression and found no evidence of benefit.
	Obsessions and compulsions	One review^48^ assessed the impact of play-based interventions on obsessions and compulsions and found no evidence of benefit.
	Overall mental health	Two reviews^25^^,^^38^ assessed the impact of play-based interventions for overall mental health, and both found evidence of benefit, although the latter showed a low effect size.
	PTSD	None
	Self-harm	None
Technology based (relevant studies: Chen, 2022^21^; Syriopoulou-Delli, 2024^43^; Weitlauf, 2014^48^)	Anxiety	Two reviews^21^^,^^48^ assessed the impact of technology-based interventions on anxiety. Chen^21^ found consistent evidence of positive effects.
	Depression	One review^48^ assessed the impact of technology-based interventions on depression and found no evidence of benefit.
	Obsessions and compulsions	None
	Overall mental health	One review^43^ assessed the impact of technology-based interventions on overall mental health and found evidence of benefit.
	PTSD	None
	Self-harm	None
Other interventions (relevant studies: Linden, 2023^8^ [arts-based]; De Vries, 2015^24^ [arts-based]; Hillman, 2020^28^ [arts-based; relaxation/mind-body/massage]; Rosenau, 2026^38^ [animal-assisted, arts-based]; Rumball, 2019^39^ [EMDR]; Vasa, 2014^45^ [sensory interventions]; Weitlauf, 2014^48^ [environmental; developmental/educational; environmental; relaxation/mind-body/massage])	There was very little evidence for a benefit of other interventions on any outcomes, although 1 review^24^ assessed the benefit of arts-based interventions for overall (as well as anxiety in 1 study) mental health and found a benefit.	

Note: ACT = acceptance and commitment therapy; CBT = cognitive-behavioral therapy; EMDR = eye movement desensitization and reprocessing; NOS = not otherwise specified; PTSD = Posttraumatic stress disorder.

As noted in Table 4 from the participatory synthesis, Rumney and MacMahon^40^ suggested that the intensity of the social skills intervention had little effect on measures of depression. In Sharma *et al.*,^41^ intervention duration did not significantly moderate CBT effects, which may reflect that most trials used 16 sessions of treatment. The key outcomes relevant to our review in the included systematic reviews were anxiety symptoms, depression symptoms, overall mental health, obsessions and compulsions. All but 1 of the reviews that used meta-analysis used standardized mean difference (SMD) approaches (including Cohen’s *d* and Hedges’ *g*), rather than conducting analysis on specific measurement scales. The exception was Warwick *et al.*,^47^ which assessed odds ratios (ORs) of complete recovery from anxiety conceptualized as a binary variable. The greatest body of evidence was available for CBT-based therapies. Among the 20 reviews that assessed interventions classified as CBT/adapted CBT/CBT-based/ACT, 4 (20%)^29^^,^^32^^,^^36^^,^^41^ solely or mostly included interventions that were specifically tailored or adapted for autistic people.

**Table 4:** Public and Patient Involvement and Engagement (PPIE) Prioritized Intervention Features

First author, year	Duration	Consistency	Setting/context	Neurodiverse involvement
Cameron, 2021^20^	Duration was not synthesized or discussed across study designs, other than clinical case reports. The duration of treatment varied across the clinical case reports, ranging from 15 individual sessions to 8 mo. Behavioral training was undertaken almost daily, whereas CBT was undertaken weekly.	Authors reported that there was a lack of information on who delivered the intervention. Two included studies reported that the intervention had been adapted for young people with autism, but it was not clear what adaptations had been made.	Not well reported. Interventions seemed to most often be delivered in clinical settings, although 1 study mentioned school and home settings.	NR
Chen, 2022^21^	Duration was not synthesized or discussed, but authors suggested future research should focus on understanding the optimal duration and intensity of each session.	Authors noted that many ASD participants enjoyed the telehealth and XR interventions and that this may in part be because scenarios and contexts presented in XR were predictable and organized, which could help participants maintain their routine.	A variety of settings. For XR interventions, the most common setting was an experiment laboratory. For telehealth interventions, the most common setting was participant’s home.	NR
D’Alo, 2021^22^	Study median duration was 8 wk (IQR: 8-22 wk).	Not relevant (pharmacological intervention).	Setting was a combination of outpatient and inpatient settings.	Key elements of the review protocol including participants, intervention, comparator, outcomes, and study design were developed by the guideline panel, including caregivers of children and adolescents with ASD.
De Crescenzo, 2020^23^	Study median duration was 12 wk (IQR: 6-52 wk).	Not relevant (food supplement intervention).	Outpatients.	Review had a guideline group comprising a multidisciplinary panel including caregivers of children and adolescents with ASD. The group helped to formulate questions for developing evidence-based health recommendations. The outcomes’ relevance to children and adolescents with ASD was also discussed.
De Vries, 2015^24^	Authors reported the ideal session frequency and duration for the intervention of music works was increments of 30-60 min at a time 4-5 times a week.	Authors reported that participants preferred environments that were predictable and known to them.	Home and school environments were the main settings used.	NR
Francis, 2022^25^	Frequencies of interventions ranged from once a month to 4 times a week, and the total duration of the interventions ranged from 100 min to 240 h.	Authors noted that the person delivering the intervention has a key role (typically a play partner to the child or a facilitator), although no evidence of their impact on outcome was reported.	Settings varied from school to home to outside of school. In the last-mentioned case, exact locations were not reported in the original studies.	NR
Gregus, 2023^26^	Authors reported that the duration of the CBT interventions ranged from 5.25 to 108 h with an average of 20.13 h. The meta-regression indicated that different treatment dosages did not result in significantly different effect sizes.	Most common adaptations to interventions included the use of visual aids, emotional labeling, social skills training, and similar structure between treatment sessions. However, there was no evidence reported on how effective these modifications were.	Most settings were clinical or not reported, with a small number at school or online.	Content analysis showed 6 studies had stakeholder involvement in CBT program creation (total 28).
Gupta, 2023^27^	Duration not synthesized or discussed.	Not relevant (pharmacological intervention).	NR	NR
Hillman, 2020^28^	Authors stated that it was unclear what effect flexibility around the number or length of sessions had on the results of this review, but discussed that it may have had an effect.	Authors noted that many interventions had been modified specifically for use with ASD populations, such as replacing group sessions with one-on-one treatment sessions, increasing the amount of time dedicated to engagement with the therapist, increasing the number of sessions dedicated to emotion recognition training, adapting activities, or incorporating clients’ special interests into treatment. However, it was not possible to identify what effect, if any, these modifications had on outcomes.	Settings were mostly clinical settings, with a small number of school-based and home-based settings.	Authors discussed involving people with ASD in design and modification of interventions planned for them, including those targeting anxiety.
James, 2020^29^	Amount of therapist contact time ranged from <10 h (16 studies) to >20 hours (23 studies). The remaining interventions involved between 10 and 20 h of therapist contact time. No differences were found between these groups.	Authors noted that most of the CBT programs had been modified to make them appropriate for children with ASD, such as by including social stories, social coaching, visual aids, and structured worksheets. However, it was not possible to identify which, if any, of these modifications may have improved outcomes.	Settings were mostly clinical outpatient facilities, with some schools. There was no clear evidence of advantage for any setting.	NR
Kose, 2018^30^	Number of sessions of CBT ranged from 6 to 17.4 over 9-21 wk; duration of sessions ranged from 35 min to 2 h.	Authors described in detail the type of modifications used, such as parental involvement, increased use of visuals, personalized treatment metaphors, self-monitoring, positive reinforcement, and use of clear language and instructions.	Mostly clinical settings, although some sessions delivered at home or, in 1 instance, at the beach or in the park.	NR
Kreslins, 2015^31^	Duration of interventions varied between 6 and 16 sessions, and the length of each session was 60-120 min. Authors discussed that having a flexible number and length of sessions may be useful treatment adaptations.	Authors noted that modification trends for CBT programs included using visual aids, incorporating child-specific interests into the intervention, using highly structured sessions, and having a flexible number and length of sessions. They also noted the importance of parental involvement.	NR	NR
Lee, 2024^32^	Duration of each session ranged from 5 to 150 min, with the weekly frequency of sessions ranging from 1 to 3 sessions per week. The entire duration of the interventions ranged from 3 wk to 6 mo, or 80-2,340 min. The total number of sessions ranged from 4 to 42 sessions. No significant differences were found based on different durations of treatment.	NR	Most interventions were delivered in the home, with a few in clinics or community centers. Interventions were mostly delivered in person, with some online. The authors noted that it may be beneficial to study effects of culturally responsive interventions that are delivered in settings, such as schools, clinics, or community settings, to promote generalization of skills.	NR
Linden, 2023^8^	NR	Interventions were divided into adapted and nonadapted. However, because of the uncertainty found in the available evidence, the authors stated that they were unable to recommend a particular modality of CBT over another.	NR	The research team included both autistic and nonautistic researchers and lay members. They had input into all stages of the project, including development of the grant submission, study design, and drafting/dissemination of the study. The authors held focus groups with autistic people to establish prioritization of outcomes with autistic lay members and autistic researchers.
Liu, 2024^33^	Authors reported that session duration (5-120 min/session), frequency (once a week to 7 times/week), total sessions (1-144 sessions), and total duration (5-16,680 min) varied across studies. Physical activity–induced benefit for overall mental health was moderated by frequency, total sessions, and total duration. Authors suggested this indicated that interventions with higher frequency (1-6 times/wk), more total sessions (1-144 sessions), or longer total duration (5-7,200 min) generated greater benefits.	NR	Nearly all the settings were in the field, with just 2 in clinical settings.	NR
Loftus, 2023^34^	Duration of the interventions varied considerably among studies, from 2 wk to 1.7 y. The 2 most common lengths of the interventions were 9 wk and 4 wk. When comparing these 2 lengths, similar results were observed. Authors discussed that further research is necessary to determine the impact that intervention frequency and duration has on outcomes.	Authors noted that about half of the studies included modifications, such as the use of simplified language, visual prompts, focus on relevant situations such as social interactions, use of concrete (rather than abstract) language, and clearly detailing each session before commencement for predictability. There was not sufficient evidence show what impact these changes had on outcomes.	Most studies were in a clinical setting, some at home or at school. The authors reported that there was no notable evidence of variance in the efficacy of interventions across settings.	Authors noted that the development of a manualized mindfulness-based intervention—codesigned specifically to address common issues experienced by the target population, including anxiety, social skill deficits, and aggressive behaviors—would enhance intervention fidelity and accessibility.
Perihan, 2020^35^	Study directly analyzed the moderator effect of treatment length. The short-term intervention category included using a CBT protocol <12 wk. Five studies used a standard term CBT protocol of 12-14 wk, and the long-term intervention category comprised studies with a protocol ≥16 wk. Treatment effects associated with short-term interventions were significantly weaker than those obtained in standard-term and long-term interventions. Some studies had also aimed to increase the efficacy of interventions in a short period of time (eg, 1 wk) by extending the contact time with clients and found that extending sessions was required rather than having more contact time overall. No statistically significance difference in intervention effect sizes between standard-term and long-term interventions was obtained.	Authors noted the need for modification of CBT protocols, but did not describe the modifications made in the included studies (beyond 2 moderators: parental involvement and length of intervention).	NR	NR
Perihan, 2022^36^	Treatment length varied from 6 to 32 wk with 60- to 90-min sessions. Authors discussed that the implementation of the clinical programs during the year (32 wk with 90-min sessions) may not be an option for most schools, as well as families, because of a lack of resources.	NR	School-based or a home-school collaboration model.	NR
Riis, 2025^37^	NR	NR	Not specified beyond any setting involving physical activity.	Authors remarked that none of the studies included a community engagement component to provide autistic people an opportunity to help shape the intervention or to solicit feedback on the intervention.
Rosenau, 2026^38^	NR	Authors discuss how it can be helpful to consider individual need when deciding on practice elements to include in an intervention. Also, that more research is needed to guide this.	NR	NR
Rumball, 2019^39^	Number of sessions ranged from 4 to 43, with a median of 10.5 sessions. Authors suggested modifications to support features of ASD, such as allowing longer session durations or more sessions to give individuals time to process and verbalize their experiences.	Only a minority of interventions reported that they had been modified to support features of ASD, including allowing longer session durations or more sessions to give individuals time to process and verbalize their experiences, simplifying language, and reducing the use of metaphors.	A variety of mostly treatment-seeking settings.	NR
Rumney, 2017^40^	Treatment time was reported but not synthesized. There was some suggestion that the intensity of the intervention had little effect on measures of depression. Intensive treatment of 145 h over a period of 6 wk did not have any significant effect on depression levels, whereas significant reductions in levels of depression were observed for interventions with a far lower treatment time of 21 h and 30 h.	No specific discussion of modification of therapies was presented, though it was noted that skills that specifically teach children and young people with ASD how to cope with social anxiety could be an important addition to anxiety interventions in future research.	A variety of settings, including clinics and classrooms, as well as online methods.	NR
Sharma, 2021^41^	Number of CBT treatment sessions ranged between 5 and 20, most commonly 16 sessions, typically occurring on a weekly basis. Authors reported that the duration of CBT was not found to be a contributing factor, but that this likely reflected most trials including a similar number of sessions.	Authors discussed the importance of adapted CBT protocols for autistic youth. Modifications were categorized into 3 areas: additions to treatment protocols to better accommodate the needs of autistic people (eg, allowing clients not to make eye contact), leaving things out (eg, focus on core beliefs), and modifying conventional practice (eg, diversifying communication techniques).	NR	NR
Simione, 2024^42^	NR	NR	Various settings, including many school settings.	NR
Syriopoulou-Delli, 2024^43^	NR	NR	NR	NR
Ung, 2015^44^	Duration of CBT sessions ranged from 60 to 120 min, and trial periods lasted from 6 to 32 wk (mean = 14.79 wk). Authors suggested that the extended period of CBT sessions was associated with more robust effects, as participants may have had more time to practice skills learned in treatment sessions.	NR	NR	NR
Vasa, 2014^45^	For pharmacological studies, duration of treatment was variable across studies (6 wk to 15 mo). For nonpharmacological studies, duration of CBT ranged from 6 to 16 wk.	Authors noted that CBT sessions were often modified. All CBT protocols included the standard CBT components, but with adaptations that might include the use of visual supports and concrete language, as well as modules to address special interests, social skills, and emotion regulation.	NR	NR
Wang, 2021^46^	NR	NR	NR	NR
Warwick, 2017^47^	Number and length of treatment sessions varied considerably across studies, but no study involved <10 h of treatment. Treatment duration varied from 14 to 24 h.	NR	A variety of settings, including research environments, clinical services, community settings, and schools	NR
Weitlauf, 2014^48^	Duration of treatment and follow-up was generally short. Few studies provided data on long-term outcomes after cessation of treatment (follow-up varied from weeks to years).	Authors noted that CBT has been adapted for children with ASD, eg, the use of more visual aids and structured worksheets, increased focus on relaxation and exposure, simplification, and decreased emphasis on cognitive components of the treatment.	A variety of settings, including clinical and research settings, community, residential, and private practice settings.	There was public stakeholder involvement in topic nomination, drafting key questions, providing input on key issues (such as inclusion/exclusion criteria). A draft report was made available for public comment.
Wichers, 2023^49^	Duration of interventions ranged from 6 to 40 wk.	Authors noted that some of the CBT interventions had been modified, such as with visual aids, implementation of social skills training in the treatment, increased parent involvement (for children), prolonged duration of treatment, and more gradual buildup of exposure elements.	NR	NR

Note: ASD = autism spectrum disorder; CBT = cognitive-behavioral therapy; IQR = interquartile range; NR = not reported; XR = extended reality.

Multiple reviews on CBT supported a benefit on anxiety, whereas 4 reviews supported a benefit on depression^20^^,^^26^^,^^48^^,^^49^ and overall mental health,^32^^,^^38^^,^^43^^,^^46^ and 2 reviews supported a benefit on obsessions and compulsions.^30^^,^^48^ When discarding reviews appraised as low and critically low in quality, evidence remained in support of CBT for reducing anxiety, but indicated that effects for depression and longer-term anxiety outcomes are more uncertain. Two reviews^32^^,^^38^ supported a benefit for behavioral therapies on overall mental health, although small effect sizes were highlighted,^38^ and the single moderate-quality review^8^ for this intervention type indicated very low certainty of evidence.

Considering findings from meta-analyses, 7 meta-analyses^8^^,^^31^^,^^35^^,^^41^^,^^44^^,^^47^^,^^49^ assessed the potential benefit of CBT for anxiety, all finding a statistically significant benefit. Effect sizes were typically moderate (eg, Hedges’ *g* = −0.66 in Perihan *et al.*^35^ and Hedges’ *g* = −0.70 in Wichers *et al.*^49^), although Sharma *et al.*^41^ found a large effect size for clinician-rated symptoms (Hedges’ *g* = 0.88) and smaller effect sizes for parent-reported anxiety (Hedges’ *g* = 0.40) and child-reported anxiety (Hedges’ *g* = 0.25). Sharma *et al.*^41^ also noted that the extent of benefit was not maintained at the longest follow-up point. Two meta-analyses assessed the potential benefit of CBT on depression: Gregus^26^ found a statistically significant benefit (SMD = −0.33 [95% CI −0.44, −0.23]), whereas Linden *et al.*^8^ did not find evidence of a statistically significant benefit for either adapted group CBT (SMD = 0.31 [95% CI −2.27, 3.00]) or individual CBT (SMD = −0.32 [95% CI −4.01, 3.40]). One meta-analysis^38^ assessed the potential benefit of CBT on overall mental health, showing a large effect size (Cohen’s *d* = 0.78).

Two reviews assessed mindfulness interventions, with one showing a benefit on anxiety and depression^42^ and the other showing a benefit on anxiety (review did not include depression).^34^ Two reviews^8^^,^^20^ generally supported a benefit of psychotherapy not otherwise specified on depression, although there was some uncertainty^20^ due to methodological issues with included studies (which was similarly identified in the moderate-quality review evidence^8^). Finally, 1 review^39^ found evidence of a benefit of behavioral therapy, CBT- or ACT-based therapies, and psychotherapy not otherwise specified for posttraumatic stress disorder.

There was some evidence of a benefit of pharmacological interventions, especially on anxiety outcomes.^8^^,^^22^^,^^27^^,^^41^^,^^45^^,^^48^ These positive effects were reflected in the moderate-quality review evidence for anxiety indications.^25^^,^^38^ One meta-analysis^22^ found that antipsychotics had a statistically significant effect on obsessions and compulsions (SMD = −0.30 [95% CI −0.55, −0.06], moderate certainty), but not on anxiety (SMD = −0.38 [95% CI −0.82, 0.07], very low certainty). There was limited evidence for biomedical (eg, dietary changes, use of supplements), family-based, or play-based interventions, although 2 reviews^8^^,^^31^^,^^32^^,^^36^^,^^40^^,^^48^^,^^49^ found evidence to support a benefit of play-based interventions for overall mental health. One meta-analysis^23^ showed a statistically significant benefit of dietary intervention with polyunsaturated fatty acids on anxiety (SMD = −1.01 [95% CI −1.86, −0.17]), but there was very low certainty of evidence. One meta-analysis^25^ assessed play-based interventions and found a large effect size for positive mental health outcomes (Cohen’s *d* = 1.60 [95% CI 0.37, 2.82]), but did not find a statistically significant benefit on negative mental health outcomes (Cohen’s *d* = 0.17 [95% CI= −0.04, 0.51]). Although multiple reviews^8^^,^^31^^,^^32^^,^^36^^,^^40^^,^^48^^,^^49^ supported a benefit of social skills interventions on anxiety and depression outcomes, these positive effects were generally reflected in the low-quality and critically low-quality reviews—findings were uncertain for the moderate-quality review data (due to high risk of bias in the evaluated studies).^8^ One meta-analysis^33^ showed a benefit of physical activity interventions on overall mental health (Hedges’ *g* = 0.67 [95% CI 0.50, 0.85]). Evidence for other interventions was limited.

### Equity Outcomes

Equity-relevant findings are shown in Table 2. This table outlines findings for both effectiveness and implementation outcomes, as well as the notable evidence gaps for most intervention types and equity characteristics.

There was limited research dedicated to evaluating equity-relevant themes at review level. An exception to this was the study by Lee *et al.*,^32^ which examined the efficacy of culturally responsive interventions—ie, original interventions that had been modified to appropriately meet the needs and preferences of diverse groups and communities (eg, social skills programs that tailored activities and linguistics to local contexts). Here, results revealed a beneficial effect for positive mental health outcomes across both child and parent measures, comparable to nonmodified autism interventions. From a neurodevelopmental perspective, several reviews sought to examine populations with co-occurring conditions or disabilities (eg, obsessive-compulsive disorder,^30^ intellectual disabilities,^20^ developmental language disorder^25^). As with Lee *et al.*,^32^ these studies addressed equity-relevant themes through targeted search and selection criteria, which preferentially identified research for their selected topic or population. However, there were no such reviews dedicated to other equity-relevant characteristics. Moreover, there are instances where targeted selection criteria would have disproportionately weakened evidence for certain groups (eg, by excluding data from individuals with co-occurring conditions).

Therefore, most equity-relevant themes presented in Table 2 relate to findings from within-review analyses—ie, synthesis of trial-level data undertaken as part of more general intervention evaluations. We identified targeted or planned analyses relating to place of residence,^29^ race/ethnicity,^47^ age,^31^^,^^41^ gender/sex,^41^^,^^45^ socioeconomic status,^47^ and disability.^29^^,^^30^^,^^43^^,^^45^ The majority of findings in these cases related to concerns from narrative syntheses about a lack of data or representation from specific populations (eg, females, ethnic minorities, people from low- or middle-income countries, and people with sociocognitive difficulties).

Nonetheless, 2 reviews did mention some equity-related issues. The first review performed quantitative moderator analyses to investigate age- and gender-related differences.^41^ The authors found that CBT was more efficacious for younger children (for clinician and parent ratings) and when delivered as individual therapy (for clinician ratings), but that there was no moderating effect regarding the number of sessions and of the gender of the recipients (although they did note that females are generally underrepresented in autism research, impacting the power of an analysis to detect a true effect). The second review highlighted (but did not investigate in any detail) equity-relevant themes in relation to implementation factors, concerning economic costs (for delivering and accessing technology-based services), system infrastructure (eg, e-communication capabilities in rural areas), and digital literacy skills.^21^

The RAG was consulted on the equity analysis findings to share their perspectives. Multiple members of the group highlighted that socioeconomic factors are highly important. This equity-relevant characteristic relates to not only accessing mental health interventions with the opportunities of private care, but also the ability to engage with interventions that could be affected by parental socioeconomic factors. Members of the group agreed that parents with less education and financial stability may find it more difficult to be informed advocates for their children, and this could affect accessing and engaging with mental health interventions.

Some members of the group also drew from their lived experience to discuss the lack of representation of autistic girls and women, which was considered concerning. This was discussed as a particularly important limitation because group members noted that autistic girls can present differently and often struggle with comorbid mental health conditions. Therefore, omitting girls in studies of mental health interventions not only means missing a significant portion of the autistic community, but also may mean that the investigated interventions are not generalizable to the autistic female population. Other equity characteristics mentioned in the RAG included religion, as religious beliefs about mental health or certain therapies may introduce barriers to interventions, and physical disabilities, which could also affect access.

### Participatory Synthesis Outcomes

The RAG identified several review characteristics that were important to them, including treatment duration, consistency, setting/context, and neurodiverse involvement. These have been extracted in Table 4.

RAG members felt strongly that neurodiverse people must be involved in the process of producing interventions that are tailored to them, as members described the neurodiverse mindset to be wholly different. Neglecting to involve neurodiverse people may lead to findings and recommendations being rejected by the community. We therefore investigated whether neurodiverse involvement occurred within the included reviews. Most of the reviews neither described that they had discussed interventions with people with lived experience of neurodiversity nor extracted data on neurodiverse involvement. However, 4 reviews^8^^,^^22^^,^^23^^,^^48^ involved PPIE in the research process, and 1 review^26^ found that 6 of 28 of its included studies had stakeholder involvement in the creation of CBT programs. Even with some involvement, our collaborators noted that without a diverse range of experiences and presentations, interventions may not be effective for the heterogeneous ASD population.

The setting or context of an intervention was also considered an important characteristic by our RAG. Our RAG members explained that some autistic people are more comfortable in certain environments or are simply unable to access others. Some settings depended on intervention (eg, physical activity interventions often took place outside), but the most common settings were clinical environments. This was followed by school- and home-based contexts. Only 1 review^34^ discussed variance of efficacy across intervention settings, finding that there were no notable effects. Further, 13 reviews did not specify details about settings at all.

Linked to setting is the consistency of an intervention, which was discussed in the context of routine, intervention deliverer, and familiarity of location. Two studies^21^^,^^24^ discussed that the more predictable or familiar the environments, the more preferrable to participants. This finding was echoed by some group members. Of the included reviews, 10 mentioned adaptations that were made to interventions to make them appropriate for ASD populations, but it was unclear whether these would improve the consistency of interventions. The RAG emphasized that keeping the same intervention deliverer and building a relationship is key to a successful intervention, but acknowledged that it would be difficult to study this in a way that mirrors real-world services.

Finally, duration was considered as an important intervention factor for the RAG group. This included both the duration of individual sessions and the overall length of the intervention. Longer sessions, particularly in the case of talking-based therapies, were seen as mentally draining and potentially unproductive or even unhelpful by RAG members, considering potential attentional or social communication difficulties. When the length of sessions was reported, these varied considerably from 5 minutes to more than 2 hours, with most reviews reporting sessions in the range of 60 to 120 minutes. The total intervention length also varied considerably, from 2 weeks to more than a year. One study had a primary focus on the moderating effect of treatment length, reporting that treatment effects were significantly weaker in short-term protocols (12 weeks or less) than standard-term (14 weeks) or long-term (16 weeks or more) protocols. This matched the RAG consensus that longer intervention lengths were more likely to be effective. Overall, in all aspects of interventions, including setting, consistency, and duration, agency for the individual is critical to collaborators. Group members reported that the ability to choose to end a session or have the intervention take place at a chosen location can be the difference between success and failure.

## Discussion

### Quantity, Quality, and Included Interventions in the Evidence

We undertook an umbrella review of mental health interventions for autistic children and adolescents, working closely with public collaborators to synthesize the evidence and identify equity-relevant aspects of interventions. After an exhaustive search, we identified 31 relevant systematic reviews, of which the majority were rated as being of low or critically low quality, signaling potential challenges with the robustness of methods used to synthesize relevant evidence for this population. Though a range of possible interventions was addressed across included reviews, the intervention with the best evidence was CBT, which was identified as effective for each of the mental health outcomes relevant in this review. Importantly, this conclusion was overall robust to the quality of included systematic reviews, though more robust reviews did temper positive conclusions somewhat by drawing clearer attention to limitations in the evidence base. Other interventions included mindfulness, social skills training, and pharmacological therapies, as well as more general reviews of behavioral interventions. Evidence for effectiveness was less consistent or clear-cut in these areas. No systematic review with a focus on prevention was retrieved.

Throughout the evidence base in general, we noted that the same pattern repeatedly emerged of clinician-reported outcomes showing the greatest benefit, with parent-reported outcomes showing less benefit and child-reported outcomes showing the least benefit. This pattern of data requires careful consideration, as prevalent concerns about the suitability and acceptability of mental health interventions can be expressed by autistic people (eg, for CBT, behavioral therapies), and the optimization of future programs should thus be guided by the perspectives and experiences of these individuals.^50^ Several of the reviews described how interventions were modified to ensure they were suitable for autistic participants. Examples given included visual aids, concrete language (ie, fewer metaphors), incorporation of child-specific interests into the intervention, and having a flexible number and length of sessions. However, evidence remains lacking on the impact of such modifications on outcomes.

### Equity Impacts and Participatory Synthesis

A major focus of this umbrella review was identification of equity dimensions of synthesized evidence and consultation with our RAG on the salience and importance of these dimensions. This is important as autistic children and adolescents who belong to (other) underserved or seldom-heard groups may face multiplicative barriers to accessing effective mental health intervention. With very few exceptions, included reviews did not address equity dimensions of interventions in depth. Our RAG noted with particular concern that included reviews were largely silent with respect to socioeconomic status of families and individuals as a barrier to access. Members similarly concerned with the absence of a sex- or gender-based analysis given the underrepresentation of autistic women and girls in intervention trials. RAG members also noted the importance of co-occurring disability, which was an area that was relatively well covered in existing reviews (although it was not uncommon for individuals with low IQ to be excluded, and reviews often highlighted a lack of data from children with high cognitive and/or social communication difficulties).

A broader point of equity relates to the near silence in included reviews on the effectiveness, or even relevance, of interventions in LMICs vs high-income countries. In high-income countries, well-established health care infrastructures with access to resources and trained professionals are the norm. Although interventions synthesized in included reviews have demonstrated effectiveness within high-resource settings, their success does not automatically translate to LMICs, where health care systems, cultural norms, and socioeconomic conditions differ significantly.

A key factor limiting generalizability is the contextual disparity between high-income countries and LMICs. For example, CBT, which was well evidenced in existing reviews, may not align with local beliefs or caregiving practices in LMICs. These cultural differences necessitate substantial adaptation to ensure that interventions are culturally relevant and resonate with families and practitioners in diverse settings.

### Future Research

The field would benefit from reviews that are expansive in terms of intervention type, to support comparisons between different approaches; attentive to equity dimensions, in terms of populations included in relevant trials, demonstrated moderation of effect within relevant trials, and differences in effect in ecological meta-regressions between trials; and attentive to equity-promoting features of interventions. For example, RAG members commented on coproduction, delivery setting, delivery context and consistency, and the importance of user input and control in service provision. However, equity in intervention design is not straightforward and requires considerable attention to context. For example, and as our RAG members noted, online or home-based provision may be a more inclusive mode of delivery for autistic people who struggle with certain sensory stimuli or with transport and mobility barriers. At the same time, this may exclude service users benefiting from face-to-face support or for whom the home environment is not conducive to intervention.

As a broader point, future research will require trialists to include diverse populations in primary studies as the foundation of developing mental health interventions that work for all autistic children and young people. This should include trials in LMIC contexts. Without carefully considering and adapting interventions to local contexts, there is a risk that their implementation in LMICs not only may be ineffective, but also could inadvertently widen existing disparities in access and quality of care. To address this, future research must prioritize context-specific adaptation and validation, actively involving local communities in the codesign of interventions and evaluation frameworks. By broadening the geographic and socioeconomic scope of research and focusing on local applicability, it becomes possible to build a more inclusive and globally relevant evidence base for autism interventions.

Finally, our approach to evidence synthesis was led by a participatory ethos, and our findings benefited from the input of lived experience contributors and researchers. Both participatory research and patient and public involvement and engagement have been part of the research lexicon for some time now. In the United Kingdom, research inclusion is now a condition of funding in applied health and care research.^51^ Even so, certain groups who are more likely to experience specific barriers to research involvement are underrepresented in these efforts and are particularly underrepresented in evidence synthesis used to inform policy. Moreover, the extent to which autistic people have been able to meaningfully shape the research agenda and its implementation has traditionally been inadequate,^52^ and participatory involvement of autistic people is often restricted (eg, stakeholders are often given limited consultation roles and remain recipients of the research, as opposed to being actively involved as partners with devolved power). This work has demonstrated the clear benefits of engaging lived experience expertise in evidence synthesis, including to identify equity determinants of particular importance to autistic children and young people.

Although included studies included mostly participants from the target population, some included youth up to approximately 25 years of age. It is possible that older participants within these studies could experience autism differently than adolescents. Although tackling this research question is challenging, given the breadth of the research and complexity of drawing and defining equity and effectiveness outcomes, the inclusion of a research team including lived experience of being an autistic person and/or a parent of an autistic child, along with guidance from our RAG, helped us ensure this review was informed by neurodiverse perspectives. Lived experience input would have been strengthened by including an RAG with more experience of cultural and socioeconomic differences. Finally, our search was undertaken to a high standard, and our approach to reviewing followed recommended guidance. However, there is always a risk that relevant reviews were not retrieved or that a different set of reviewers may have applied inclusion and exclusion criteria in a different way, with possible implications for interpretation. As an umbrella review, some primary studies feature in more than 1 included systematic review; however, this issue is reduced by the fact that we used a narrative synthesis rather than a meta-analytic approach, as well as the diversity of research questions asked by included systematic reviews.

Mental health interventions for autistic children and adolescents remain an important priority, with at least 1 well-evidenced treatment modality (CBT). However, current evidence does not highlight the equity dimensions that might plausibly impact the effectiveness and efficiency of these interventions, both in terms of population groups and in terms of intervention design. Future work should take an intervention-wide, equity-deep approach to synthesis and to the design and analysis of primary trials.

## CRediT authorship contribution statement

**Alan Lovell:** Writing – review & editing, Writing – original draft, Project administration, Methodology, Formal analysis. **Kieran Becker:** Writing – original draft, Methodology, Investigation. **Maxwell S. Barnish:** Writing – original draft, Methodology, Formal analysis. **Tom Arthur:** Writing – original draft, Methodology, Formal analysis. **Joelle Kirby:** Formal analysis. **Helen F. Dodd:** Writing – review & editing, Methodology, Data curation. **Vijaya Raghavan:** Writing – review & editing, Methodology. **G.J. Melendez-Torres:** Writing – review & editing, Writing – original draft, Supervision, Methodology, Funding acquisition, Conceptualization. **Anna Price:** Writing – review & editing, Supervision, Methodology, Investigation.
