# Supplementary material for: Umbrella Review: Mental Health Interventions for Autistic Children and Adolescents: An Overview of Systematic Reviews and Participatory Equity Synthesis
Source: JAACAP Open. 2026 Feb 26;4(3):399–427. doi: 10.1016/j.jaacop.2026.02.004 (PMC13221812; doi:10.1016/j.jaacop.2026.02.004)
Supplement: Supplementary Data [file mmc1.docx]

# Supplement 1

# Supplemental materials for: Mental Health Interventions for Autistic Children and Adolescents: An Overview of Systematic Reviews and Participatory Equity Synthesis

## Searches

Database(s): **Ovid MEDLINE(R) ALL**1946 to November 07, 2024
Search Strategy:

| **#** | **Searches** | **Results** |
| --- | --- | --- |
| 1 | exp Autism Spectrum Disorder/ | 46634 |
| 2 | (autis* or asperger* or asd or asc or "demand avoid*" or PDA or EDA).ti,ab,kf. | 122209 |
| 3 | (("pervasive developmental disorder" or PDD or PDDNOS or neurodive* or (cognitiv* or neurolog* or neurodev*)) adj3 (diver* or variab*)).ti,ab,kf. | 8978 |
| 4 | or/1-3 | 134072 |
| 5 | exp mental health services/ | 109007 |
| 6 | ((mental* or psycho* or emotional or mood or depress*) adj3 (unwell or distress* or ill* or disorder* or trauma* or condition* or health or wellbeing or "well being")).ti,ab,kf. | 581951 |
| 7 | exp Mental Disorders/ or mental health/ | 1563666 |
| 8 | (schizo* or hebephreni* or oligophreni* or psychotic* or psychosis or psychoses).tw. | 219640 |
| 9 | (paranoia or paranoid disorders or psychotic disorders or psychosis).tw. | 53624 |
| 10 | ((bipolar or bi polar) adj5 (disorder* or depress*)).tw. | 44746 |
| 11 | (hypomania* or mania* or manic*).tw. | 23110 |
| 12 | (depress* or anxiet* or OCD or compulsive* or obsessive* or post-traumatic* or ptsd or "post traumatic" or bipolar* or psychosis or psychotic or schizophrenia or schizoaffective or "borderline personality" or "eating disorder*" or "self harm" or self-harm or suicide or suicidal).ti,kf. | 567934 |
| 13 | (depress* or anxiet* or OCD or compulsive* or obsessive* or post-traumatic* or ptsd or "post traumatic" or bipolar* or psychosis or psychotic or schizophrenia or schizoaffective or "borderline personality" or "eating disorder*" or "self harm" or self-harm or suicide or suicidal).ab. /freq=2 | 627794 |
| 14 | or/5-13 | 2240792 |
| 15 | (systematic review or meta-analysis).pt. | 367235 |
| 16 | meta-analysis/ or systematic review/ or systematic reviews as topic/ or meta-analysis as topic/ or "meta analysis (topic)"/ or "systematic review (topic)"/ or exp technology assessment, biomedical/ or network meta-analysis/ | 410092 |
| 17 | ((systematic* adj3 (review* or overview*)) or (methodologic* adj3 (review* or overview*))).ti,ab,kf. | 388154 |
| 18 | ((quantitative adj3 (review* or overview* or synthes*)) or (research adj3 (integrati* or overview*))).ti,ab,kf. | 18503 |
| 19 | ((integrative adj3 (review* or overview*)) or (collaborative adj3 (review* or overview*)) or (pool* adj3 analy*)).ti,ab,kf. | 44441 |
| 20 | (data synthes* or data extraction* or data abstraction*).ti,ab,kf. | 47988 |
| 21 | (handsearch* or hand search*).ti,ab,kf. | 11815 |
| 22 | (mantel haenszel or peto or der simonian or dersimonian or fixed effect* or latin square*).ti,ab,kf. | 39868 |
| 23 | (met analy* or metanaly* or technology assessment* or HTA or HTAs or technology overview* or technology appraisal*).ti,ab,kf. | 13548 |
| 24 | (meta regression* or metaregression*).ti,ab,kf. | 17390 |
| 25 | (meta-analy* or metaanaly* or systematic review* or biomedical technology assessment* or bio-medical technology assessment*).mp,hw. | 547493 |
| 26 | (medline or cochrane or pubmed or medlars or embase or cinahl).ti,ab,hw. | 403783 |
| 27 | (cochrane or (health adj2 technology assessment) or evidence report).jw. | 22164 |
| 28 | (comparative adj3 (efficacy or effectiveness)).ti,ab,kf. | 19932 |
| 29 | (outcomes research or relative effectiveness).ti,ab,kf. | 12042 |
| 30 | ((indirect or indirect treatment or mixed-treatment or bayesian) adj3 comparison*).ti,ab,kf. | 4807 |
| 31 | (meta-analysis or systematic review).mp. | 512876 |
| 32 | (multi* adj3 treatment adj3 comparison*).ti,ab,kf. | 320 |
| 33 | (mixed adj3 treatment adj3 (meta-analy* or metaanaly*)).ti,ab,kf. | 184 |
| 34 | umbrella review*.ti,ab,kf. | 2338 |
| 35 | (multi* adj2 paramet* adj2 evidence adj2 synthesis).ti,ab,kf. | 15 |
| 36 | (multiparamet* adj2 evidence adj2 synthesis).ti,ab,kf. | 19 |
| 37 | (multi-paramet* adj2 evidence adj2 synthesis).ti,ab,kf. | 13 |
| 38 | or/15-37 | 792031 |
| 39 | exp adolescent/ or exp child/ or exp infant/ or (infant disease* or childhood disease*).ti,ab,kf. or (adolescen* or babies or baby or boy? or boyfriend or boyhood or girlfriend or girlhood or child* or girl? or infan* or juvenil* or kid? or minors or minors* or neonat* or neo-nat* or newborn* or new-born* or paediatric* or peadiatric* or pediatric* or perinat* or preschool* or puber* or pubescen* or school* or teen* or toddler? or underage? or under-age? or youth*).ti,ab,kf. or (pediatric* or paediatric* or infan* or child* or adolescen* or young).jn,jw. or (pediatric* or paediatric* or infan* or child* or adolescen* or young).in. | 6022404 |
| 40 | 4 and 14 and 38 and 39 | 2465 |
| 41 | limit 40 to yr="2014 -Current" | 2063 |

Database(s): **Embase**1974 to 2024 November 07
Search Strategy:

| **#** | **Searches** | **Results** |
| --- | --- | --- |
| 1 | exp autism/ | 105298 |
| 2 | (autis* or asperger* or asd or asc or "demand avoid*" or PDA or EDA).ti,ab,kf. | 164065 |
| 3 | (("pervasive developmental disorder" or PDD or PDDNOS or neurodive* or (cognitiv* or neurolog* or neurodev*)) adj3 (diver* or variab*)).ti,ab,kf. | 12058 |
| 4 | or/1-3 | 198723 |
| 5 | exp mental health care/ or mental health/ or psychological well-being/ | 406154 |
| 6 | ((mental* or psycho* or emotional or mood or depress*) adj3 (unwell or distress* or ill* or disorder* or trauma* or condition* or health or wellbeing or "well being")).ti,ab,kf. | 741486 |
| 7 | schizophrenia spectrum disorder/ | 2735 |
| 8 | (schizo* or hebephreni* or oligophreni* or psychotic* or psychosis or psychoses).tw. | 289140 |
| 9 | exp Psychotic Disorders/ | 343458 |
| 10 | (paranoia or paranoid disorders or psychotic disorders or psychosis).tw. | 81032 |
| 11 | exp mood disorder/ | 733891 |
| 12 | ((bipolar or bi polar) adj5 (disorder* or depress*)).tw. | 68702 |
| 13 | (hypomania* or mania* or manic*).tw. | 32250 |
| 14 | exp depression/ | 682070 |
| 15 | exp anxiety disorder/ | 349088 |
| 16 | (depress* or anxiet* or OCD or compulsive* or obsessive* or post-traumatic* or ptsd or "post traumatic" or bipolar* or psychosis or psychotic or schizophrenia or schizoaffective or "borderline personality" or "eating disorder*" or "self harm" or self-harm or suicide or suicidal).ti,kf. | 740297 |
| 17 | (depress* or anxiet* or OCD or compulsive* or obsessive* or post-traumatic* or ptsd or "post traumatic" or bipolar* or psychosis or psychotic or schizophrenia or schizoaffective or "borderline personality" or "eating disorder*" or "self harm" or self-harm or suicide or suicidal).ab. /freq=2 | 865842 |
| 18 | or/5-17 | 1989782 |
| 19 | (systematic review or meta-analysis).pt. | 0 |
| 20 | meta-analysis/ or systematic review/ or systematic reviews as topic/ or meta-analysis as topic/ or "meta analysis (topic)"/ or "systematic review (topic)"/ or exp technology assessment, biomedical/ or network meta-analysis/ | 716469 |
| 21 | ((systematic* adj3 (review* or overview*)) or (methodologic* adj3 (review* or overview*))).ti,ab,kf. | 468033 |
| 22 | ((quantitative adj3 (review* or overview* or synthes*)) or (research adj3 (integrati* or overview*))).ti,ab,kf. | 21191 |
| 23 | ((integrative adj3 (review* or overview*)) or (collaborative adj3 (review* or overview*)) or (pool* adj3 analy*)).ti,ab,kf. | 62246 |
| 24 | (data synthes* or data extraction* or data abstraction*).ti,ab,kf. | 58126 |
| 25 | (handsearch* or hand search*).ti,ab,kf. | 14403 |
| 26 | (mantel haenszel or peto or der simonian or dersimonian or fixed effect* or latin square*).ti,ab,kf. | 52590 |
| 27 | (met analy* or metanaly* or technology assessment* or HTA or HTAs or technology overview* or technology appraisal*).ti,ab,kf. | 22860 |
| 28 | (meta regression* or metaregression*).ti,ab,kf. | 21127 |
| 29 | (meta-analy* or metaanaly* or systematic review* or biomedical technology assessment* or bio-medical technology assessment*).mp,hw. | 847368 |
| 30 | (medline or cochrane or pubmed or medlars or embase or cinahl).ti,ab,hw. | 522429 |
| 31 | (cochrane or (health adj2 technology assessment) or evidence report).jw. | 32518 |
| 32 | (comparative adj3 (efficacy or effectiveness)).ti,ab,kf. | 29152 |
| 33 | (outcomes research or relative effectiveness).ti,ab,kf. | 17336 |
| 34 | ((indirect or indirect treatment or mixed-treatment or bayesian) adj3 comparison*).ti,ab,kf. | 8395 |
| 35 | (meta-analysis or systematic review).mp. | 802121 |
| 36 | (multi* adj3 treatment adj3 comparison*).ti,ab,kf. | 459 |
| 37 | (mixed adj3 treatment adj3 (meta-analy* or metaanaly*)).ti,ab,kf. | 266 |
| 38 | umbrella review*.ti,ab,kf. | 2449 |
| 39 | (multi* adj2 paramet* adj2 evidence adj2 synthesis).ti,ab,kf. | 36 |
| 40 | (multiparamet* adj2 evidence adj2 synthesis).ti,ab,kf. | 22 |
| 41 | (multi-paramet* adj2 evidence adj2 synthesis).ti,ab,kf. | 31 |
| 42 | or/19-41 | 1130721 |
| 43 | exp adolescence/ or exp adolescent/ or exp child/ or exp childhood disease/ or exp infant disease/ or (adolescen* or babies or baby or boy? or boyfriend or boyhood or girlfriend or girlhood or child* or girl? or infan* or juvenil* or juvenile* or kid? or minors or minors* or neonat* or neonat* or neo-nata* or newborn* or new-born* or paediatric* or peadiatric* or pediatric* or perinat* or preschool* or puber* or pubescen* or school or school child* or school* or schoolchild* or schoolchild* or teen* or toddler? or underage? or under-age? or youth*).ti,ab,kw. | 6572273 |
| 44 | 4 and 18 and 42 and 43 | 1920 |
| 45 | limit 44 to yr="2014 -Current" | 1633 |

Database(s): **APA PsycInfo**1806 to October 2024 Week 5
Search Strategy:

| **#** | **Searches** | **Results** |
| --- | --- | --- |
| 1 | exp Autism Spectrum Disorders/ | 60413 |
| 2 | (autis* or asperger* or asd or asc or "demand avoid*" or PDA or EDA).ti,ab,id. | 75172 |
| 3 | (("pervasive developmental disorder" or PDD or PDDNOS or neurodive* or (cognitiv* or neurolog* or neurodev*)) adj3 (diver* or variab*)).ti,ab,id. | 9058 |
| 4 | or/1-3 | 84870 |
| 5 | mental health services/ | 41812 |
| 6 | ((mental* or psycho* or emotional or mood or depress*) adj3 (unwell or distress* or ill* or disorder* or trauma* or condition* or health or wellbeing or "well being")).ti,ab,id. | 582283 |
| 7 | exp Mental Disorders/ or exp mental health/ or exp well being/ | 1266044 |
| 8 | (schizo* or hebephreni* or oligophreni* or psychotic* or psychosis or psychoses).tw. | 203696 |
| 9 | (paranoia or paranoid disorders or psychotic disorders or psychosis).tw. | 63153 |
| 10 | ((bipolar or bi polar) adj5 (disorder* or depress*)).tw. | 41609 |
| 11 | (hypomania* or mania* or manic*).tw. | 24115 |
| 12 | (depress* or anxiet* or OCD or compulsive* or obsessive* or post-traumatic* or ptsd or "post traumatic" or bipolar* or psychosis or psychotic or schizophrenia or schizoaffective or "borderline personality" or "eating disorder*" or "self harm" or self-harm or suicide or suicidal).ti,id. | 534566 |
| 13 | (depress* or anxiet* or OCD or compulsive* or obsessive* or post-traumatic* or ptsd or "post traumatic" or bipolar* or psychosis or psychotic or schizophrenia or schizoaffective or "borderline personality" or "eating disorder*" or "self harm" or self-harm or suicide or suicidal).ab. /freq=2 | 541826 |
| 14 | or/5-13 | 1630860 |
| 15 | (systematic review or meta-analysis).pt. | 0 |
| 16 | meta-analysis/ or systematic review/ or systematic reviews as topic/ or meta-analysis as topic/ or "meta analysis (topic)"/ or "systematic review (topic)"/ or exp technology assessment, biomedical/ or network meta-analysis/ | 6243 |
| 17 | ((systematic* adj3 (review* or overview*)) or (methodologic* adj3 (review* or overview*))).ti,ab. | 65634 |
| 18 | ((quantitative adj3 (review* or overview* or synthes*)) or (research adj3 (integrati* or overview*))).ti,ab. | 12039 |
| 19 | ((integrative adj3 (review* or overview*)) or (collaborative adj3 (review* or overview*)) or (pool* adj3 analy*)).ti,ab. | 7319 |
| 20 | (data synthes* or data extraction* or data abstraction*).ti,ab. | 4632 |
| 21 | (handsearch* or hand search*).ti,ab. | 1739 |
| 22 | (mantel haenszel or peto or der simonian or dersimonian or fixed effect* or latin square*).ti,ab. | 7005 |
| 23 | (met analy* or metanaly* or technology assessment* or HTA or HTAs or technology overview* or technology appraisal*).ti,ab. | 1165 |
| 24 | (meta regression* or metaregression*).ti,ab. | 3176 |
| 25 | (meta-analy* or metaanaly* or systematic review* or biomedical technology assessment* or bio-medical technology assessment*).mp,hw. | 96332 |
| 26 | (medline or cochrane or pubmed or medlars or embase or cinahl).ti,ab,hw. | 42887 |
| 27 | (cochrane or (health adj2 technology assessment) or evidence report).jw. | 0 |
| 28 | (comparative adj3 (efficacy or effectiveness)).ti,ab. | 2563 |
| 29 | (outcomes research or relative effectiveness).ti,ab. | 4146 |
| 30 | ((indirect or indirect treatment or mixed-treatment or bayesian) adj3 comparison*).ti,ab. | 576 |
| 31 | (meta-analysis or systematic review).mp. | 85129 |
| 32 | (multi* adj3 treatment adj3 comparison*).ti,ab. | 52 |
| 33 | (mixed adj3 treatment adj3 (meta-analy* or metaanaly*)).ti,ab. | 19 |
| 34 | umbrella review*.ti,ab. | 410 |
| 35 | (multi* adj2 paramet* adj2 evidence adj2 synthesis).ti,ab. | 2 |
| 36 | (multiparamet* adj2 evidence adj2 synthesis).ti,ab. | 6 |
| 37 | (multi-paramet* adj2 evidence adj2 synthesis).ti,ab. | 2 |
| 38 | or/15-37 | 146895 |
| 39 | exp adolescent/ or exp child/ or exp infant/ or (infant disease* or childhood disease*).ti,ab. or (adolescen* or babies or baby or boy? or boyfriend or boyhood or girlfriend or girlhood or child* or girl? or infan* or juvenil* or kid? or minors or minors* or neonat* or neo-nat* or newborn* or new-born* or paediatric* or peadiatric* or pediatric* or perinat* or preschool* or puber* or pubescen* or school* or teen* or toddler? or underage? or under-age? or youth*).ti,ab. | 1405288 |
| 40 | 4 and 14 and 38 and 39 | 1771 |
| 41 | limit 40 to yr="2014 -Current" | 1447 |

**CINAHL**

| # | Query | Limits | Results |
| --- | --- | --- | --- |
| S5 | (TI ( (adolescen* or babies or baby or boy? or boyfriend or boyhood or girlfriend or girlhood or child* or girl? or infan* or juvenil* or kid? or minors or minors* or neonat* or neo-nat* or newborn* or new-born* or paediatric* or peadiatric* or pediatric* or perinat* or preschool* or puber* or pubescen* or school* or teen* or toddler? or underage? or under-age? or youth*) ) OR AB ( (adolescen* or babies or baby or boy? or boyfriend or boyhood or girlfriend or girlhood or child* or girl? or infan* or juvenil* or kid? or minors or minors* or neonat* or neo-nat* or newborn* or new-born* or paediatric* or peadiatric* or pediatric* or perinat* or preschool* or puber* or pubescen* or school* or teen* or toddler? or underage? or under-age? or youth*) ) OR MW ( adolescent or child or infant )) AND (S1 AND S2 AND S3 AND S4) | Publication Date: 20140101-20241231 | 1,019 |
| S4 | TI ( (adolescen* or babies or baby or boy? or boyfriend or boyhood or girlfriend or girlhood or child* or girl? or infan* or juvenil* or kid? or minors or minors* or neonat* or neo-nat* or newborn* or new-born* or paediatric* or peadiatric* or pediatric* or perinat* or preschool* or puber* or pubescen* or school* or teen* or toddler? or underage? or under-age? or youth*) ) OR AB ( (adolescen* or babies or baby or boy? or boyfriend or boyhood or girlfriend or girlhood or child* or girl? or infan* or juvenil* or kid? or minors or minors* or neonat* or neo-nat* or newborn* or new-born* or paediatric* or peadiatric* or pediatric* or perinat* or preschool* or puber* or pubescen* or school* or teen* or toddler? or underage? or under-age? or youth*) ) OR MW ( adolescent or child or infant ) |  | 1,398,951 |
| S3 | ( (MH "Systematic Review") OR (MH "Meta Analysis") OR (MH "Meta Synthesis") ) OR TI ( (meta-analy* or metaanaly* or systematic review* or biomedical technology assessment* or bio-medical technology assessment*) ) OR AB ( (meta-analy* or metaanaly* or systematic review* or biomedical technology assessment* or bio-medical technology assessment*) ) OR TI ( ((systematic* N3 (review* or overview*)) or (methodologic* N3 (review* or overview*))) ) OR AB ( ((systematic* N3 (review* or overview*)) or (methodologic* N3 (review* or overview*))) ) OR TI ( ((quantitative N3 (review* or overview* or synthes*)) or (research N3 (integrati* or overview*))) ) OR AB ( ((quantitative N3 (review* or overview* or synthes*)) or (research N3 (integrati* or overview*))) ) OR AB ( (medline or cochrane or pubmed or medlars or embase or cinahl) ) |  | 306,784 |
| S2 | (MH "Mental Health Services+") OR TI ( ((mental* or psycho* or emotional or mood or depress*) N3 (unwell or distress* or ill* or disorder* or trauma* or condition* or health or wellbeing or "well being")) ) OR AB ( ((mental* or psycho* or emotional or mood or depress*) N3 (ill* or disorder* or trauma* or condition* or health or wellbeing or "well being")) ) OR (MH "Mental disorders+") OR (MH "Mental health") OR AB ( (schizo* or hebephreni* or oligophreni* or psychotic* or psychosis or psychoses) ) OR AB ( (paranoia or paranoid disorders or psychotic disorders or psychosis) ) OR AB ( ((bipolar or bi polar) N5 (disorder* or depress*)) ) OR AB ( (hypomania* or mania* or manic*) ) OR TI ( (depress* or anxiet* or OCD or compulsive* or obsessive* or post-traumatic* or ptsd or "post traumatic" or bipolar* or psychosis or psychotic or schizophrenia or schizoaffective or "borderline personality" or "eating disorder*" or "self harm" or self-harm or suicide or suicidal) ) |  | 944,826 |
| S1 | ( (MH "Autism Spectrum Disorder") OR TI (autis* or asperger* or asd or asc or "demand avoid*" or PDA or EDA or "pervasive developmental disorder" or PDD or PDDNOS or neurodive*) OR AB (autis* or asperger* or asd or asc or "demand avoid*" or PDA or EDA or "pervasive developmental disorder" or PDD or PDDNOS or neurodive*) ) |  | 49,302 |

## Table S1: List of inclusion and exclusion criteria

| **Domain** | **Inclusion criteria** | **Exclusion criteria** |
| --- | --- | --- |
| Population | - Autistic children and adolescents (up to 19 years of age) - Eligible diagnoses include Autistic Spectrum Conditions/Autism Spectrum Disorder, Asperger's syndrome and Pervasive Development Disorder Not Otherwise Specified - Neurodiverse populations more broadly where reviews include a focus on autistic children and adolescents, or children and adolescents with autistic traits | - Adults - Mixed adult and child or adolescent populations where results for children and adolescents are not presented separately |
| Intervention | - Mental health interventions, defined as interventions evaluated for prevention or treatment of mental health problems - Interventions implemented with parents as carers are eligible if outcomes relating to children and adolescents’ mental health are included | - Interventions not focused on the treatment of mental health problems |
| Comparator | - Any comparator intervention | - No comparator intervention |
| Outcomes | - For reviews focusing on effectiveness, any outcome related to mental health-related symptoms, diagnoses or health states - For reviews focusing on implementation, data relating to acceptability, feasibility and mechanisms of interventions and their implementation | - Reviews focusing on ‘symptoms of autism’ as an outcome or capturing quality of life impacts more broadly - Irritability, disruptive behaviors or other aspects of autism that were not considered mental health symptoms |
| Study design | - Systematic reviews published as full texts in the English language (2014 onwards) - Aimed at assessing effectiveness or implementation of interventions | - Non-systematic reviews (that do not use specific search strategies, state inclusion and exclusion criteria or synthesize identified evidence) - Scoping reviews - Reviews published before 2014 - Reviews published in languages other than English - Reviews not aimed at assessing effectiveness or implementation of interventions - Reviews aimed at assessing a broader range of outcomes, where the synthesis of effectiveness or implementation is not separable |

“Health state” refers to the category of in this case mental wellness to which an individual belongs in an analysis, e.g. “healthy”, “sick” or “deceased”.

## Table S2: List of excluded studies

| # | First author (year) | Title | Reason for exclusion |
| --- | --- | --- | --- |
| 1 | Adams (2019) | Anxiety in children with autism at school: A systematic review | Outcome |
| 2 | Aithal (2021) | A Systematic Review of the Contribution of Dance Movement Psychotherapy Towards the Well-Being of Children With Autism Spectrum Disorders | Outcome |
| 3 | Alabdulkareem (2022) | A Systematic Review of Research on Robot-Assisted Therapy for Children with Autism | Outcome |
| 4 | AlBhaisi (2022) | Effectiveness of psychological techniques in dental management for children with autism spectrum disorder: a systematic literature review | Setting |
| 5 | Ali (2024) | Adiponectin blood levels and autism spectrum disorders: a systematic review | Intervention |
| 6 | Allison (2023) | A Survey of Virtual Reality Interventions for Autistic Spectrum Disorder Therapy: A Neuroscience Perspective | Publication type |
| 7 | Alvares (2021) | Use of probiotics in pediatric patients with autism spectrum disorder: a systematic review | Outcome |
| 8 | Ameis (2018) | Systematic review and guide to management of core and psychiatric symptoms in youth with autism | Study type |
| 9 | Archibald (2014) | Mapping the waters: A scoping review of the use of visual arts in pediatric populations with health conditions | Publication type |
| 10 | Ash (2017) | Physical activity interventions for children with social, emotional, and behavioral disabilities-a systematic review | Population |
| 11 | Bailey (2022) | Virtual reality and augmented reality for children, adolescents, and adults with communication disability and neurodevelopmental disorders: A systematic review | Outcome |
| 12 | Barry (2020) | A scoping review of the barriers and facilitators to the implementation of interventions in autism education | Intervention |
| 13 | Bennewith (2024) | Sublime and extended reality experiences to enhance emotional wellbeing for autistic people: A state of the art review and narrative synthesis | Outcome |
| 14 | Berenguer (2020) | Exploring the Impact of Augmented Reality in Children and Adolescents with Autism Spectrum Disorder: A Systematic Review | Outcome |
| 15 | Bertelli (2015) | Multimodal antidepressants in the treatment of anxiety and depressive disorders in persons with neurodevelopmental disorders | Publication type |
| 16 | Boaden (2020) | Antidepressants in Children and Adolescents: Meta-Review of Efficacy, Tolerability and Suicidality in Acute Treatment | Outcome |
| 17 | Bottema-Beutel (2023) | An evaluation of intervention research for transition-age autistic youth | Outcome |
| 18 | Bowman-Perrott (2023) | Peer-Mediated Interventions for Students with Intellectual and Developmental Disabilities: A Systematic Review of Reviews of Social and Behavioral Outcomes | Outcome |
| 19 | Brooker (2021) | Psychological therapies in autism | Publication type |
| 20 | Byrd (2024) | Physiotherapeutic Interventions in Child and Adolescent Mental Health Services: The evidence for who, what and why | Publication type |
| 21 | Camilleri (2022) | Autism spectrum disorder and social story research: A scoping study of published, peer-reviewed literature reviews | Publication type |
| 22 | Camino-Alarcon (2024) | A Systematic Review of Treatment for Children with Autism Spectrum Disorder: The Sensory Processing and Sensory Integration Approach | Outcome |
| 23 | Charry-Sanchez (2018) | Effectiveness of Animal-Assisted Therapy in the Pediatric Population: Systematic Review and Meta-Analysis of Controlled Studies | Population |
| 24 | Cherewick (2024) | Neurodiversity in practice: A conceptual model of autistic strengths and potential mechanisms of change to support positive mental health and wellbeing in autistic children and adolescents | Intervention |
| 25 | Cleary (2023) | Putting Cats on the Spectrum: A Scoping Review of the Role of Cats in Therapy and Companionship for Autistic Adults and Children | Population |
| 26 | Cleary (2024) | A Scoping Review of Equine-Assisted Therapies on the Mental Health and Well-Being of Autistic Children and Adolescents: Exploring the Possibilities | Publication type |
| 27 | Conrad (2021) | Parent-Mediated Interventions for Children and Adolescents With Autism Spectrum Disorders: A Systematic Review and Meta-Analysis | Outcome |
| 28 | da Rosa (2024) | Exploring clozapine use in severe psychiatric symptoms associated with autism spectrum disorder: A scoping review | Publication type |
| 29 | Davis (2015) | Animal assisted interventions for children with autism spectrum disorder: A systematic review | Outcome |
| 30 | De Filippis (2016) | Treatment of autism spectrum disorder in children and adolescents | Study type |
| 31 | de Nocker (2023) | Using telehealth to provide interventions for children with ASD: A systematic review | Outcome |
| 32 | Deb (2020) | The effectiveness of parent training for children with autism spectrum disorder: a systematic review and meta-analyses | Outcome |
| 33 | Delli (2018) | Review of interventions for the management of anxiety symptoms in children with ASD | Publication type |
| 34 | Diaz (2024) | Family systemic therapy: intervention in autism spectrum disorder | Publication type |
| 35 | Dickson (2021) | Correction to: A Systematic Review of Mental Health Interventions for ASD: Characterizing Interventions, Intervention Adaptations, and Implementation Outcomes | Publication type |
| 36 | Dickson (2021) | A Systematic Review of Mental Health Interventions for ASD: Characterizing Interventions, Intervention Adaptations, and Implementation Outcomes | Outcome |
| 37 | Dickson (2022) | Short report: A quantitative methodological review of participant characteristics in the literature testing mental health interventions for youth with autism spectrum disorder | Intervention |
| 38 | Donath (2024) | [The Effect of Exercise Therapy on Adolescent Mental Health: A Systematic Review with Practical Example] | Language |
| 39 | Dyer (2020) | Outcomes for patients when using service dogs and animal-assisted therapy dogs to address autism, dementia, and posttraumatic stress disorder: A systematic review | Outcome |
| 40 | Edwards (2020) | Performance and visual arts-based programs for children with disabilities: a scoping review focusing on psychosocial outcomes | Study type |
| 41 | Edwards (2022) | Cognitive control training for children with anxiety and depression: A systematic review | Population |
| 42 | Elliott (2021) | Behavioral and cognitive behavioural therapy for obsessive compulsive disorder (OCD) in individuals with autism spectrum disorder (ASD) | Population |
| 43 | Fan (2023) | Nature-Based Interventions for Autistic Children: A Systematic Review and Meta-Analysis | Outcome |
| 44 | Ferguson (2024) | The Effects of Yoga Practices on Self-Regulation in Children with Autism Spectrum Disorder: A Systematic Review | Publication type |
| 45 | Flujas-Contreras (2023) | Effectiveness of psychological interventions in Autism Spectrum Disorder (ASD): A systematic review of meta-analyses and systematic reviews | Language |
| 46 | Garcia-Vazquez (2023) | Social cognition interventions for adolescents with autism spectrum disorder. A systematic review | Outcome |
| 47 | Gassner (2021) | Effectiveness Of Music Therapy For Autism Spectrum Disorder, Dementia, Depression, Insomnia, And Schizophrenia | Publication type |
| 48 | Gassner (2022) | Effectiveness of music therapy for autism spectrum disorder, dementia, depression, insomnia and schizophrenia: update of systematic reviews | Outcome |
| 49 | Goyal (2023) | Evidence-based analysis of multi-pronged approaches for education and behavior management of autistic patients in a dental setting | Setting |
| 50 | Greenlee (2018) | Family level processes associated with outcomes for individuals with autism spectrum disorder: A scoping review | Study type |
| 51 | Gupta (2023) | 6.32 N-Methyl-D-Aspartate (NMDA) Receptor Antagonist and ASD: A Systematic Review | Publication type |
| 52 | Hangul (2022) | Use of complementary and alternative therapies in autism spectrum disorder | Language |
| 53 | Hartley (2019) | Mindfulness for Children and Adults with Autism Spectrum Disorder and Their Caregivers: A Meta-analysis | Outcome |
| 54 | Hartley (2022) | Barriers and facilitators to engaging individuals and families with autism spectrum disorder in mindfulness and acceptance-based therapies: a meta-synthesis | Population |
| 55 | Hatoum (2024) | Applications and efficacy of radically open dialectical behavior therapy (RO DBT): A systematic review of the literature | Population |
| 56 | Haupt (2021) | A systematic review and meta-analysis of cognitive-behavioral therapy for children and adolescents with autism and anxiety | Publication type |
| 57 | Hillman (2024) | SPECIAL ISSUE: Use of Heart Rate Variability Biofeedback for Reducing Anxiety Among College Students Diagnosed with Autism Spectrum Disorder: A Brief Literature Review | Full text availability |
| 58 | Ho (2015) | Cognitive behavioural approach for children with autism spectrum disorder: A literature review | Outcome |
| 59 | Hoagwood (2017) | Animal-Assisted Therapies for Youth with or at risk for Mental Health Problems: A Systematic Review | Outcome |
| 60 | Hollis (2017) | Annual Research Review: Digital health interventions for children and young people with mental health problems - a systematic and meta-review | Outcome |
| 61 | Hothi (2022) | Evaluated interventions addressing developmental transitions for youth with mental health disorders: a meta-analysis | Outcome |
| 62 | Hourston (2017) | Autism and Mind-Body Therapies: A Systematic Review | Population |
| 63 | Hume (2021) | Evidence-Based Practices for Children, Youth, and Young Adults with Autism: Third Generation Review | Outcome |
| 64 | Husgen (2022) | A systematic review of dog-assisted therapy in children with behavioural and developmental disorders | Outcome |
| 65 | Kester (2018) | Cognitive behavior therapy to treat anxiety among children with autism spectrum disorders: A systematic review | Outcome |
| 66 | Khan (2019) | The Effectiveness of Web-Based Interventions Delivered to Children and Young People With Neurodevelopmental Disorders: Systematic Review and Meta-Analysis | Outcome |
| 67 | Khan (2020) | The therapeutic role of Cannabidiol in mental health: a systematic review | Population |
| 68 | Kokol (2020) | Serious Game-based Intervention for Children with Developmental Disabilities | Outcome |
| 69 | Lake (2019) | Systematic review comparing efficacy and effectiveness trials of cognitive behavioural therapy among youth with autism | Publication type |
| 70 | Lake (2020) | Considering efficacy and effectiveness trials of cognitive behavioral therapy among youth with autism: A systematic review | Outcome |
| 71 | Li (2020) | Efficacy and Safety of Fluoxetine in Autism Spectrum Disorder: A Meta-analysis | Publication type |
| 72 | Li (2023) | Effectiveness of acceptance and commitment therapy-based interventions for improving the psychological health of parents of children with special health care needs: A systematic review and meta-analysis | Population |
| 73 | Limbu (2022) | Randomised controlled trials of mood stabilisers for people with autism spectrum disorder: systematic review and meta-analysis | Outcome |
| 74 | Line (2024) | A meta-analysis of parent-inclusive child therapy interventions for decreasing symptomatology | Population |
| 75 | Littell (2021) | Multisystemic TherapyÂ® for social, emotional, and behavioural problems in youth age 10 to 17: An updated systematic review and meta-analysis | Outcome |
| 76 | Liu (2024) | Web-based physical activity programs for individuals with autism spectrum disorder and their parents: a systematic review and meta-analysis | Population |
| 77 | Loizou (2024) | Approaches to improving mental healthcare for autistic people: systematic review | Population |
| 78 | Long (2022) | Yoga: The Symptomatic, Behavioral, and Emotional Impact on Children with a Life-Long Illness | Population |
| 79 | Maniram (2022) | Pharmacological management of core symptoms and comorbidities of autism spectrum disorder in children and adolescents: A systematic review | Outcome |
| 80 | Manley (2024) | Supporting the health and wellbeing of trans autistic school-aged youth: a systematic literature review | Outcome |
| 81 | Mao (2023) | The effectiveness of mindfulness-based interventions for ruminative thinking: A systematic review and meta-analysis of randomized controlled trials | Population |
| 82 | Marini (2023) | Deep brain stimulation for autism spectrum disorder | Publication type |
| 83 | Marino (2021) | Third time's the charm or three strikes you're out? An updated review of the efficacy of dolphin-assisted therapy for autism and developmental disabilities | Outcome |
| 84 | Martínez-Vérez (2024) | Interventions through Art Therapy and Music Therapy in Autism Spectrum Disorder, ADHD, Language Disorders, and Learning Disabilities in Pediatric-Aged Children: A Systematic Review | Outcome |
| 85 | Maujean (2015) | A systematic review of randomized controlled trials of animal-assisted therapy on psychosocial outcomes | Population |
| 86 | Mazon (2019) | Effectiveness and usability of technology-based interventions for children and adolescents with ASD: A systematic review of reliability, consistency, generalization and durability related to the effects of intervention | Outcome |
| 87 | McCrossin (2023) | Measuring family outcomes in parenting programs for children with neurodisabilities: a scoping review | Publication type |
| 88 | Mechler (2015) | Effects of glutamatergic agents in the treatment of compulsivity and impulsivity in child and adolescent psychiatry: A systematic review | Publication type |
| 89 | Melbye (2020) | Smartphone-Based Self-Monitoring, Treatment, and Automatically Generated Data in Children, Adolescents, and Young Adults With Psychiatric Disorders: Systematic Review | Population |
| 90 | Melvin (2022) | A systematic review of in-patient psychiatric care for people with intellectual disabilities and/or autism: effectiveness, patient safety and experience | Population |
| 91 | Menezes (2020) | Treatment of Depression in Individuals with Autism Spectrum Disorder: A Systematic Review | Population |
| 92 | Menezes (2022) | Treatment of anxiety in autistic adults: A systematic review | Population |
| 93 | Mesa-Gresa (2018) | Effectiveness of Virtual Reality for Children and Adolescents with Autism Spectrum Disorder: An Evidence-Based Systematic Review | Outcome |
| 94 | Micai (2024) | Effectiveness, implementation settings, and research priorities of telemedicine-delivered interventions for children and adolescents with autism spectrum disorder: A systematic review | Outcome |
| 95 | Mittal (2024) | Effect of immersive virtual reality-based training on cognitive, social, and emotional skills in children and adolescents with autism spectrum disorder: A meta-analysis of randomized controlled trials | Outcome |
| 96 | Moo (2024) | Family-centered creative arts therapies for children with autism: A configurative systematic review | Outcome |
| 97 | Moon (2020) | Mobile device applications and treatment of autism spectrum disorder: a systematic review and meta-analysis of effectiveness | Outcome |
| 98 | Muszkat (2016) | Transcranial Direct Current Stimulation in Child and Adolescent Psychiatry | Outcome |
| 99 | Novak (2016) | Effectiveness of occupational therapy intervention for children with disabilities: Systematic review | Publication type |
| 100 | Ong (2024) | Psychopharmacological management of obsessive-compulsive behavior in children and adolescents with autism spectrum disorders: a narrative review | Study type |
| 101 | Pemovska (2024) | Approaches to improving mental health care for autistic children and young people: a systematic review and meta-analysis | Population |
| 102 | Peng (2022) | Meta-analysis and systematic review of physical activity on neurodevelopment disorders, depression, and obesity among children and adolescents | Outcome |
| 103 | Rafiei (2023) | [Formula: see text]Is active video gaming associated with improvements in social behaviors in children with neurodevelopmental disorders: a systematic review | Outcome |
| 104 | Rehn (2023) | The effectiveness of animal-assisted therapy for children and adolescents with autism spectrum disorder: A systematic review | Outcome |
| 105 | Rosenbrock (2021) | Treatment acceptability in parent-mediated interventions: Considerations for maximizing outcomes for children with autism | Study type |
| 106 | Sagar-Ouriaghli (2018) | Propranolol for treating emotional, behavioural, autonomic dysregulation in children and adolescents with autism spectrum disorders | Outcome |
| 107 | Schweizer (2014) | Art therapy with children with Autism Spectrum Disorders: A review of clinical case descriptions on 'what works' | Study type |
| 108 | Seeber (2019) | 1.34 WEARABLE TECHNOLOGY AND ASD: A REVIEW | Publication type |
| 109 | Semple (2019) | Review: Yoga and mindfulness for youth with autism spectrum disorder: review of the current evidence | Outcome |
| 110 | Sengupta (2024) | Nurturing Well-Being: Impact of Homoeopathy on Children's Behavior | Study type |
| 111 | Shah (2024) | Role of Virtual Reality in Treating Anxiety in Child & Adolescent | Publication type |
| 112 | Shi (2016) | Meta analysis of influence of music therapy on emotion, language, behavior and social skills in children with autism | Language |
| 113 | Sopena (2023) | Eye movement desensitization and reprocessing therapy for individuals with neurodevelopmental disorders: A systematic review | Population |
| 114 | Sotulenko (2023) | 6.9 Dance Therapy for Children and Adolescents With ASD: A Scoping Review | Publication type |
| 115 | Spain (2017) | Family therapy for autism spectrum disorders | Outcome |
| 116 | Stegemann (2019) | Music Therapy and Other Music-Based Interventions in Pediatric Health Care: An Overview | Population |
| 117 | Tielsch (2015) | The Role and Impact of Animals with Pediatric Patients | Population |
| 118 | Tirupathi (2024) | Effect of Sensory Adapted Dental Environment (SADE) on physiological and behavioral parameters related to stress and anxiety in children with Autism Spectrum Disorder (ASD) undergoing dental treatment: A systematic review and meta-analysis | Setting |
| 119 | Trelles (2017) | Evidenced-based behavioral and pharmacological interventions | Publication type |
| 120 | Vancampfort (2020) | [The efficacy of physical activity on psychiatric symptoms and physical health in people with psychiatric disorders: a systematic review of recent meta-analyses] | Language |
| 121 | Vogel (2024) | Art therapy for children and adolescents with autism: A systematic review | Outcome |
| 122 | Wattanawongwan (2022) | The effectiveness of interventions targeting social-communication skills for adolescents and adults with autism spectrum disorders: a meta-analysis, quality review, and single-case analysis | Publication type |
| 123 | Welch (2022) | Use of Mobile and Wearable Artificial Intelligence in Child and Adolescent Psychiatry: Scoping Review | Study type |
| 124 | Wols (2024) | Effectiveness of applied and casual games for young people's mental health: A systematic review of randomised controlled studies | Outcome |
| 125 | Yinger (2017) | Music therapy in child and adolescent mental health treatment | Publication type |

## Table S3: List of intervention categories

| **Most common** |
| --- |
| - Behavioral/behavior training/behavior therapy/Applied Behavior Analysis (ABA) - Cognitive Behavioral Therapy (CBT)/adapted CBT/CBT-based/Acceptance and Commitment Therapy (ACT) - Mindfulness-based - Psychotherapy not otherwise specified (NOS, which does not fit into any of the above and is labelled as psychotherapy) - Pharmacological - Biomedical (e.g. diet/supplements) - Physical activity - Parent management/parent training/parent psychoeducation/family therapy - Social skills interventions - Play-based interventions - Technology based (Video games, virtual reality (VR) etc.) |
| **Rare** |
| - Animal assisted therapy - Arts-based (music, art, theatre-based interventions) - Developmental/Educational- based on developmental theories of learning - Environmental (structure, planning etc.) - TEACCH is a specific example of this - Counselling - Eye movement desensitization and reprocessing (EMDR) - Sensory interventions - Psychoeducation - Relaxation/Mind-Body/Massage |

## Table S4: Risk of Bias assessment (AMSTAR II)

| Short Title | 1. PICO components | 2. Protocol | 3. Study design explanation | 4. Search strategy | 5. Duplicate study selection | 6. Duplicate data extraction | 7. Details of excluded studies | 8. Description of included studies | 9a. RoB assessment (RCTs) | 9b. RoB assessment (NRSIs) | 10. Funding sources | 11a. Meta-analysis (RCTs) | 11b. Meta-analysis (NRSIs) | 12. MA: RoB in individual studies | 13. RoB: discussion of results | 14. Heterogeneity | 15. Publication bias | 16. Reports conflicts of interest | Overall rating |
| --- | --- | --- | --- | --- | --- | --- | --- | --- | --- | --- | --- | --- | --- | --- | --- | --- | --- | --- | --- |
| Cameron (2021) | No | Yes | No | No | Yes | No | No | Partial Yes | Yes | Yes | No | N/A | N/A | N/A | Yes | No | N/A | Yes | Critically low |
| Chen (2022) | No | No | No | Partial Yes | Yes | Yes | No | Yes | No | Yes | No | N/A | N/A | N/A | No | No | N/A | Yes | Critically low |
| D'Alo (2021) | Yes | No | Yes | Yes | Yes | Yes | Yes | Yes | Yes | N/A | Yes | Yes | N/A | Yes | Yes | Yes | Yes | Yes | Low |
| De Crescenzo (2020) | Yes | No | No | Partial Yes | Yes | Yes | Partial Yes | Yes | Yes | N/A | Yes | Yes | N/A | No | Yes | Yes | Yes | Yes | Low |
| De Vries (2015) | No | No | No | Partial Yes | No | No | No | No | N/A | No | No | N/A | N/A | N/A | No | No | N/A | No | Critically low |
| Francis (2022) | Yes | Partial Yes | Yes | Partial Yes | Yes | Yes | No | Yes | Yes | Yes | No | Yes | N/A | Yes | Yes | Yes | Yes | Yes | Low |
| Gregus (2024) | Yes | No | Yes | Yes | Yes | Yes | No | No | Yes | Partial Yes | No | Yes | No | No | Yes | Yes | Yes | No | Critically low |
| Gupta (2023) | Yes | Yes | No | Partial Yes | Yes | Yes | No | Yes | Yes | Yes | No | N/A | N/A | N/A | No | No | N/A | Yes | Critically low |
| Hillman (2020) | Yes | Yes | Yes | Yes | Yes | Yes | Yes | Yes | Yes | No | No | Yes | Yes | Yes | Yes | Yes | Yes | Yes | Low |
| James (2020) | Yes | Yes | Yes | Yes | Yes | Yes | Yes | Yes | Yes | N/A | Yes | Yes | N/A | Yes | Yes | Yes | Yes | Yes | Moderate |
| Kose (2018) | No | No | No | Partial Yes | No | No | No | Yes | No | No | No | N/A | N/A | N/A | No | Yes | N/A | Yes | Critically low |
| Kreslins (2015) | No | No | No | Partial Yes | Yes | Yes | No | Yes | Yes | N/A | No | Yes | N/A | Yes | Yes | Yes | Yes | Yes | Critically low |
| Lee (2024) | No | No | No | No | Yes | Yes | No | Yes | No | No | No | Yes | Yes | No | Yes | No | Yes | Yes | Critically low |
| Linden (2023) | Yes | Yes | Yes | Yes | Yes | Yes | Yes | Yes | Yes | N/A | Yes | Yes | N/A | Yes | Yes | Yes | Yes | Yes | Moderate |
| Liu (2024) | No | Yes | No | Partial Yes | Yes | Yes | No | Yes | Yes | Yes | No | Yes | Yes | No | Yes | Yes | Yes | Yes | Low |
| Loftus (2023) | Yes | No | Yes | Yes | Yes | Yes | No | Yes | Yes | Yes | No | N/A | N/A | N/A | Yes | Yes | N/A | Yes | Critically low |
| Perihan (2020) | No | No | Yes | Partial Yes | No | Yes | No | Partial Yes | No | No | No | Yes | No | No | No | No | Yes | Yes | Critically low |
| Perihan (2022) | No | No | No | Partial Yes | No | Yes | No | Yes | No | No | No | Yes | Yes | No | No | Yes | Yes | Yes | Critically low |
| Riis (2024) | Yes | No | No | Partial Yes | Yes | No | No | Yes | Yes | No | No | N/A | N/A | N/A | No | No | N/A | Yes | Critically low |
| Rosenau (2024) | No | No | Yes | Partial Yes | Yes | Yes | No | No | No | N/A | No | Yes | N/A | No | No | No | No | Yes | Critically low |
| Rumball (2019) | No | No | No | Partial Yes | No | No | No | Yes | Yes | Yes | No | N/A | N/A | N/A | Yes | Yes | N/A | Yes | Critically low |
| Rumney (2017) | Yes | No | No | Partial Yes | No | No | Yes | Yes | Yes | No | No | N/A | N/A | N/A | Yes | Yes | N/A | No | Critically low |
| Sharma (2021) | No | No | No | No | No | Yes | No | Partial Yes | Yes | N/A | No | Yes | N/A | No | Yes | Yes | Yes | Yes | Critically low |
| Simione (2024) | No | No | Yes | No | No | Yes | No | Partial Yes | No | Yes | No | N/A | N/A | N/A | Yes | No | N/A | Yes | Critically low |
| Syriopoulou-Delli (2024) | No | No | No | No | Yes | No | No | Partial Yes | No | No | No | N/A | N/A | N/A | No | No | N/A | Yes | Critically low |
| Ung (2015) | No | No | No | Partial Yes | No | Yes | No | Yes | No | No | No | Yes | No | No | No | Yes | Yes | Yes | Critically low |
| Vasa (2014) | Yes | No | No | No | No | No | No | Partial Yes | No | No | No | N/A | N/A | N/A | No | Yes | N/A | No | Critically low |
| Wang (2021) | No | No | Yes | Partial Yes | Yes | Yes | No | Partial Yes | Partial Yes | N/A | No | Yes | Yes | No | No | Yes | Yes | Yes | Critically low |
| Warwick (2017) | Yes | No | Yes | Yes | Yes | Yes | Yes | No | Yes | N/A | No | Yes | N/A | No | Yes | Yes | No | No | Critically low |
| Weitlauf (2014) | Yes | No | No | Partial Yes | Yes | No | Yes | Yes | Partial Yes | Yes | Yes | N/A | N/A | N/A | Yes | Yes | N/A | Yes | Low |
| Wichers (2023) | No | Yes | Yes | Partial Yes | Yes | Yes | Yes | Yes | Yes | N/A | No | Yes | N/A | No | No | Yes | Yes | Yes | Low |
